# Mining Significant Substructure Pairs for Interpreting Polypharmacology in Drug-Target Network

**DOI:** 10.1371/journal.pone.0016999

**Published:** 2011-02-23

**Authors:** Ichigaku Takigawa, Koji Tsuda, Hiroshi Mamitsuka

**Affiliations:** 1 Bioinformatics Center, Institute for Chemical Research, Kyoto University, Uji, Kyoto, Japan; 2 Computational Biology Research Center, National Institute of Advanced Industrial Science and Technology, Koto-ku, Tokyo, Japan; 3 Institute for Bioinformatics Research and Development, Japan Science and Technology Agency, Chiyoda-ku, Tokyo, Japan; 4 ERATO Minato Project, Japan Science and Technology Agency, Sapporo, Hokkaido, Japan; Griffith University, Australia

## Abstract

A current key feature in drug-target network is that drugs often bind to multiple targets, known as polypharmacology or drug promiscuity. Recent literature has indicated that relatively small fragments in both drugs and targets are crucial in forming polypharmacology. We hypothesize that principles behind polypharmacology are embedded in paired fragments in molecular graphs and amino acid sequences of drug-target interactions. We developed a fast, scalable algorithm for mining significantly co-occurring subgraph-subsequence pairs from drug-target interactions. A noteworthy feature of our approach is to capture significant paired patterns of subgraph-subsequence, while patterns of either drugs or targets only have been considered in the literature so far. Significant substructure pairs allow the grouping of drug-target interactions into clusters, covering approximately 75% of interactions containing approved drugs. These clusters were highly exclusive to each other, being statistically significant and logically implying that each cluster corresponds to a distinguished type of polypharmacology. These exclusive clusters cannot be easily obtained by using either drug or target information only but are naturally found by highlighting significant substructure pairs in drug-target interactions. These results confirm the effectiveness of our method for interpreting polypharmacology in drug-target network.

## Introduction

To understand the principles behind drug-target interactions is important for safer and more efficacious treatment of diseases. A recently-identified, key feature of drug-target networks is polypharmacology or drug promiscuity, which is currently recognized as important, because of a variety of reasons which can be summarized into roughly four: 1) Exquisitely selective drugs for a single target are likely to exhibit low clinical efficacy and be unsuccessful [1]. 2) Multi-targeted drugs have been clinically successful, particularly as dual or multiplex kinase inhibitors [2]. 3) Many approved drugs, potentially the majority of therapeutic agents, are less selective than initially thought [3]. An example is cancer drugs such as Gleevec (imatinib) and Sutent (sunitinib) that show binding promiscuity for multiple kinases [4]. 4) The robustness of biological systems can be implied by the scale-free nature of drug-target networks [5], [6]. This means that some mechanisms to compensate for dysfunction of a single protein might exist, indicating that inhibiting a single target would be therapeutically insufficient [1].

Recent analysis reveals that targets of promiscuous drugs can be observed across different families abundantly [7] and can be shared among drugs which are unrelated with each other, being in different therapeutic categories [3]. These results suggest that targets of promiscuous drugs can be dissimilar, implying that only a small part of each target is related with the principle of polypharmacology. Similarly, recent research shows that smaller drugs in molecular weight are likely to be more promiscuous [6], suggesting that small fragments in each ligand would be a key to drug promiscuity. We hypothesize that paired fragments significantly shared in drug-target pairs could be crucial factors behind polypharmacology. Drugs (or chemical compounds) can be typically represented by molecular graphs, and targets (or proteins) are by amino acid sequences. Thus some key principles of polypharmacology could be observed as paired fragments (or substructures) of molecular graphs and amino acid sequences of drug-target pairs. Currently many drug-target pairs are already known, by which we can take a data-driven approach to search substructure pairs significantly shared in the drug-target (graph-sequence) pairs. We developed a scalable and efficient algorithm for systematically exploring the substructure (subgraph-subsequence) pairs which significantly co-occur in currently available drug-target pairs. We investigated the relation of the significant substructure pairs to forming polypharmacology.

One experimental result we obtained in this analysis is clustering drug-target pairs by significant substructure pairs. Possible related work would be the analysis over a drug-target network, e.g. [8]. However to the best of our knowledge, clustering over a bipartite graph of drugs and networks has not been done yet, implying that the first attempt of clustering drug-target pairs with drug promiscuity is done by using substructure pairs in this work.

## Results and Discussion

Results and data shown in this paper are all available at the website, GRASP (GRAph-Sequence Pairs): http://www.bic.kyoto-u.ac.jp/pathway/grasp.

### Data

**Figure 1:**
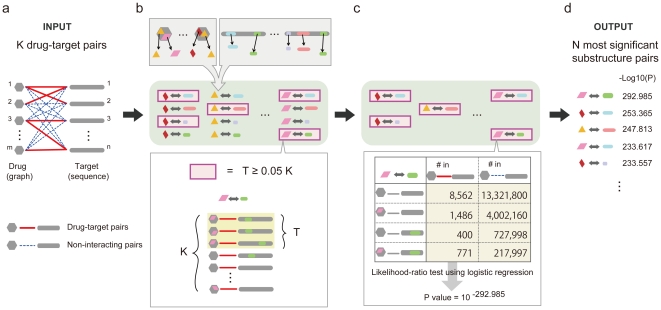
The Proposed mining Algorithm. (a) The input was *K* drug-target pairs with *m* drugs and *n* targets, and (*m x n – K*) non-interacting pairs. Practically *K* = 11,219, *m* = 4,191, *n* = 4,362, and *m x n – K* = 18,269,923. (b) All frequently co-occurring substructure pairs in *K* drug-target pairs are enumerated exhaustively, where a frequent substructure pair appeared in more than 560 drug-target pairs, i.e. 5% of 11,219 drug-target pairs. (c) The significance of each frequent substructure pair is measured by an interaction test, i.e. log-likelihood test with logistic regression, which can provide *p*-values for frequent substructure pairs to be ranked. (d) The output was *N* most significant substructure pairs, where in practice *N* = 10,000.

Our main data has two subsets: 1) 11,219 drug-target pairs containing 4,191 compounds and 4,632 targets, derived from DrugBank [9] of version 2.5 (January 29, 2009), and 2) Non-interacting pairs, corresponding to all pairs between 4,191 compounds and 4,632 targets except 11,219 drug-target pairs (Fig. 1 and Methods section). The 4,191 compounds contain 1,082 approved drugs (except those in the most minor two categories: “neutraceutical” and “withdrawn”), forming 2,723 drug-target pairs.

### Capturing significantly co-occurring substructure pairs

**Figure 2:**
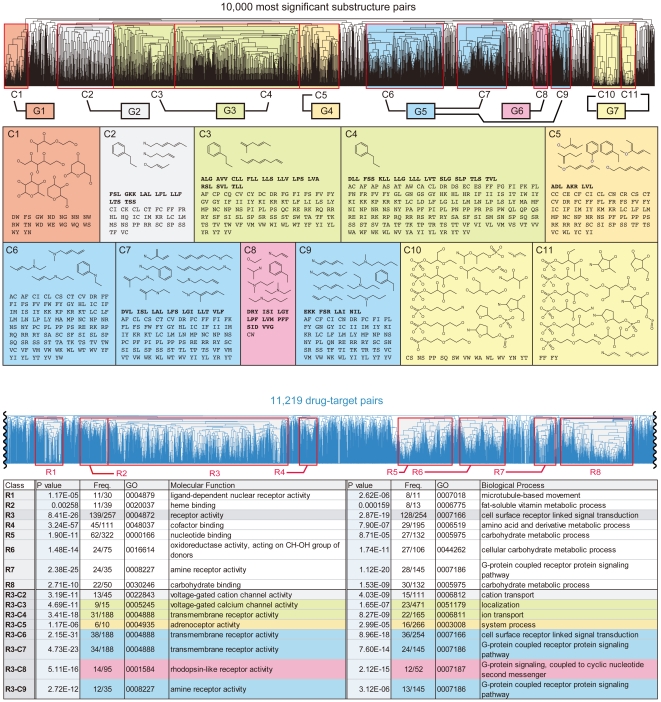
Major significant substructures and related GO terms. (a) Drug substructures and target substructures of significant substructure pairs in each of 11 clusters (7 groups), removing redundancy by taking the maximum substructures when redundancy found in each cluster. (b) The most relevant GO terms in molecular function and biological process are shown with *p*-values and the number of appearances for R1 to R8 and R3-G2 to R3-G6.

We developed a fast data-mining algorithm that has two key features:1) We exhaustively list up all frequently co-occurring pairs of substructures in drag-target pairs. 2) For each of frequent substructure pairs, we check the co-occurrence significance by a statistical interaction test that evaluates the interdependence of two substructures (Methods section, Methods S1 and Fig. 1). We obtained 41,543,488 frequent substructure pairs co-occurring in at least 5% in drug-target pairs and selected the 10,000 most significantly co-occurring substructure pairs. The significant substructure pairs had *p*-values (with no adjustment) of 10^−21.71^ to 10^−292.99^ with the average of 10^−34.73^, being selected out of approximately 4.15×10^7^ pairs and confirming the statistical significance of the substructure pairs even under the Bonferroni correction. The significant substructure pairs consist of 855 unique drug substructures (subgraphs) with the averaged molecular weight of 105.49 and the averaged size of 7.70 (hydrogen-suppressed) atoms and 6.79 bonds, and 360 unique target substructures (subsequences, being 174 with three-letter, 185 with two-letter and 1 with only one-letter) with the averaged length of 2.48 (Fig. 2a). Given significant substructure pairs, for an arbitrary compound-protein (graph-sequence) pair, we can compute a binary vector of 10,000 elements where if a significant substructure pair is included, the value of the corresponding element is 1; otherwise zero. We call this binary vector a GRASP fingerprint.

### Profiling drug-target network through significant substructure pairs

**Figure 3:**
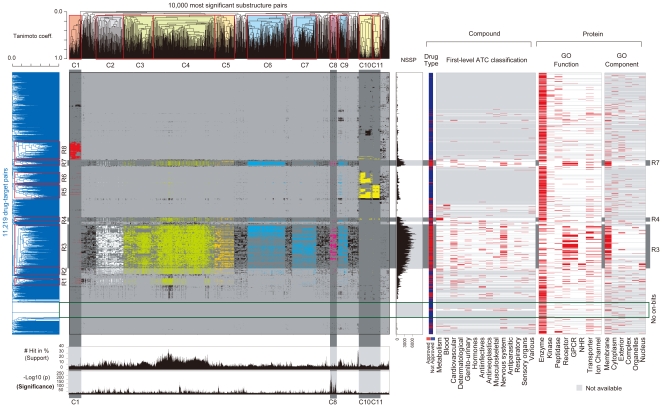
A matrix of drug-target pairs vs. significant substructure pairs. Elements are colored differently according to seven groups (G1 to G7) of significant substructure pairs. For each of drug-target pairs, the number of significant substructure pairs contained (NSSP), the 1^st^ level Anatomical Therapeutic Chemical (ATC) classification classes for the drug and gene ontology (GO) terms for the target are shown on the right-hand side of the matrix (Figure 2b for GO terms). For each of significant substructure pairs, the number of appearances in drug-target pairs (Support, shown by the ratio to all pairs) and the *p*-value (–log10(*p*)) of interaction test are shown below the matrix.

We generated a matrix of 11,219 drug-target pairs (rows) vs. 10,000 significant substructure pairs (columns) where an element takes 1 if the corresponding drug-target pair has the corresponding significant substructure pair; otherwise zero, making each row a GRASP fingerprint (Fig. 3 and Fig. S1). The number of 1s in a row ranges from zero to 7,272, the median being 226 (the first and third quartiles being 22 and 978.5, showing an asymmetric distribution) and the number of rows with no less than 226 1s being 5,613. We performed hierarchical clustering on each dimension of the matrix by using the Tanimoto coefficient (Tc) between binary vectors and then selected the major part in resultant clusters of each dimension for detailed analysis (Fig. 3 and Methods). On significant substructure pairs, we chose the 11 largest clusters (C1 to C11), covering 7,803 (78.03%) of all 10,000 and being further turned into seven groups (G1 to G7) by the substructure similarity between clusters (Fig. 2a and Fig. 3). G1 to G7 were characterized by drug substructures mainly: G1: sugar-derived drug substructures, G2: carbon skeletons with a benzene ring or nitrogen- or oxygen-containing carbon skeletons, G3: carbon skeletons with a benzene ring, G4: oxygen-containing carbon skeletons, G5: nitrogen-containing carbon skeletons, G6: drug substructures (and target substructures) related with G protein-coupled receptors (GPCRs), G7: sugar-phosphate-derived drug substructures. On drug-target pairs, we chose the most major eight clusters (R1 to R8), which covered 4,581 rows (40.83% of all 11,219; Table S1) in which the number of rows, each having no less than 226 1s, was 4,396, which was 96.0% of the 4,581 and 78.31% of the 5,613 rows with no less than 226 1s (Table S2 and Fig. S2). Some of R1 to R8 were tightly and uniquely linked to some of G1 to G7, such as R8 and G1 (Fig. 3). R1 to R8 covered 2,036 (74.77%) of the 2,723 approved-drug-containing drug-target pairs. R1 to R8 were characterized by the most relevant GO terms to targets (Fig. 2b and Table S3).

### Clustered drug-target pairs by significant substructure pairs reveal types of polypharmacology

**Figure 4:**
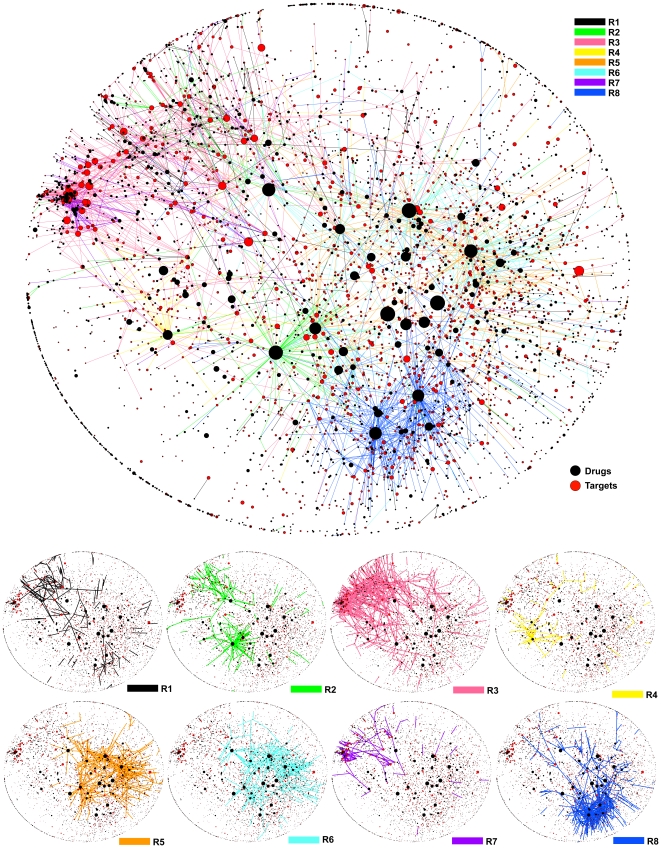
A drug-target network on interactions in R1 to R8. The edges (drug-target interactions) were colored according to R1 to R8, accompanying with 8 subnetworks, each being one of R1 to R8. The size of nodes indicates the size of degrees (or promiscuity).

Two drug-target pairs in a cluster should have a high similarity in terms of GRASP fingerprints. In fact, the average Tc of paired GRASP fingerprints in each of R1 to R8 was significantly higher than that in random clusters, each having the same number of pairs randomly selected out of the 11,219 drug-target pairs (0.243 to 0.477 while that for random clusters was 0.0374; empirical *p*-value<10^−5^; Methods and Table S4). This feature was kept for drug-target pairs with promiscuous drugs only (0.274 to 0.493 while that for random clusters was 0.0394; empirical *p*-value<10^−5^; Table S5). Drug-target pairs of polypharmacology would be divided into different types, which can be separated from each other. We hypothesize that each of R1 to R8 corresponds to a unique type of polypharmacology. To validate the hypothesis, given drugs and targets of drug-target pairs in a cluster, we measured the ratio of interactions fallen into the cluster to all interactions between them, which was significantly higher than that by random clusters (0.935 to 1.0 while that for random clusters was 0.514 to 0.610; empirical *p*-value<10^−5^; Table S6). For pairs with promiscuous drugs, again this ratio was significantly high (0.915 to 1.0 while that for random clusters was 0.498 to 0.619; empirical *p*-value<10^−5^; Table S7). As such, the exclusiveness of R1 to R8 implies that each of R1 to R8 might correspond to a unique polypharmacology type where each cluster was generated by the corresponding set of significant substructure pairs. The highly exclusive clusters cannot be found so clearly by using either drug or target information only. This can be confirmed by that drug-target pairs were clustered more clearly by GRASP fingerprints than those by compound similarity only, sequence identity only and using the sum of both (Fig. S3). In addition, the average sequence identity between drug-target pairs sharing the same drugs (i.e. promiscuous drugs) was only 0.0311, showing the diversity of targets in promiscuous drugs and implying the difficulty of clustering drug-target pairs by drug or target information only (Table S8). Interactions in R1 to R8 can be shown as a drug-target network, where edges are colored according to R1 to R8 (Fig. 4). This figure shows that each of R1 to R8, in most cases, corresponds to a dense and relatively non-overlapped subnetwork automatically. In particular, in Fig. 4, R3 can be found as an upper-left subnetwork, while R8 forms a lower-right subnetwork. We emphasize that each of these subnetworks can be explained by significant substructure pairs uniquely. This indicates that our method of mining significant substructure pairs provides with richer information than the drug-target network itself and simple clusters which can be obtained by running a graph clustering method over a drug-target network.

### Highly significant substructure pairs were specific to GPCRs

**Figure 5:**
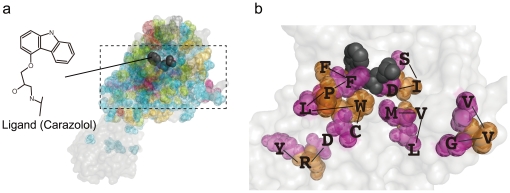
3D structure of GPCR and a ligand. (a) 3D structure of GPCR (β2 adrenergic receptor) and a ligand (Carazolol, colored black) derived from PDB, target substructure in GPCR being colored according to the corresponding group of significant substructure pairs. (b) An enlargement of (a), focusing on the binding site with target substructures including DRY (for activation), and CW, LPF, PFF, LVM and SID (for binding), all being in G6.

 Highly significant substructure pairs with the smallest *p*-values were mainly in G6 (Fig. 2), which is closely related with GPCRs, indicated by target substructures of G6, such as DRY, PFF, and CW, known short sequences of GPCRs (G6 of Fig. 3a). In fact, DRY is highly conserved in GPCRs, deeply involved with the GPCRs activation by regulating the conformational states [10], and CWxPFF is also important in ligand binding and activation [11], [12]. In addition, LPF, LVM, PFF, SID and CW form the main part of the binding pocket in the 3D structure of a GPCR, i.e. β2-adrenergic receptor bound to carazolol (Fig. 5), where ‘F’ of PFF and ‘D’ of SID are important in ligand binding from mutagenesis studies [13]. GPCRs have many conserved sequences, and it is hard to specify known target substructures in G6, such as DRY, PFF and CW, by using sequence information only, underlining the importance of focusing on both drug and target substructures. On drug substructures, endogenous ligands of adrenergic GPCRs such as adrenaline, noradrenaline, dopamine, and serotonin having propylamine (-C-C-C-N) and a benzene ring, were typical drug substructures of G6.

### Significant substructure pairs are less likely to appear in random compound-protein pairs

**Figure 6:**
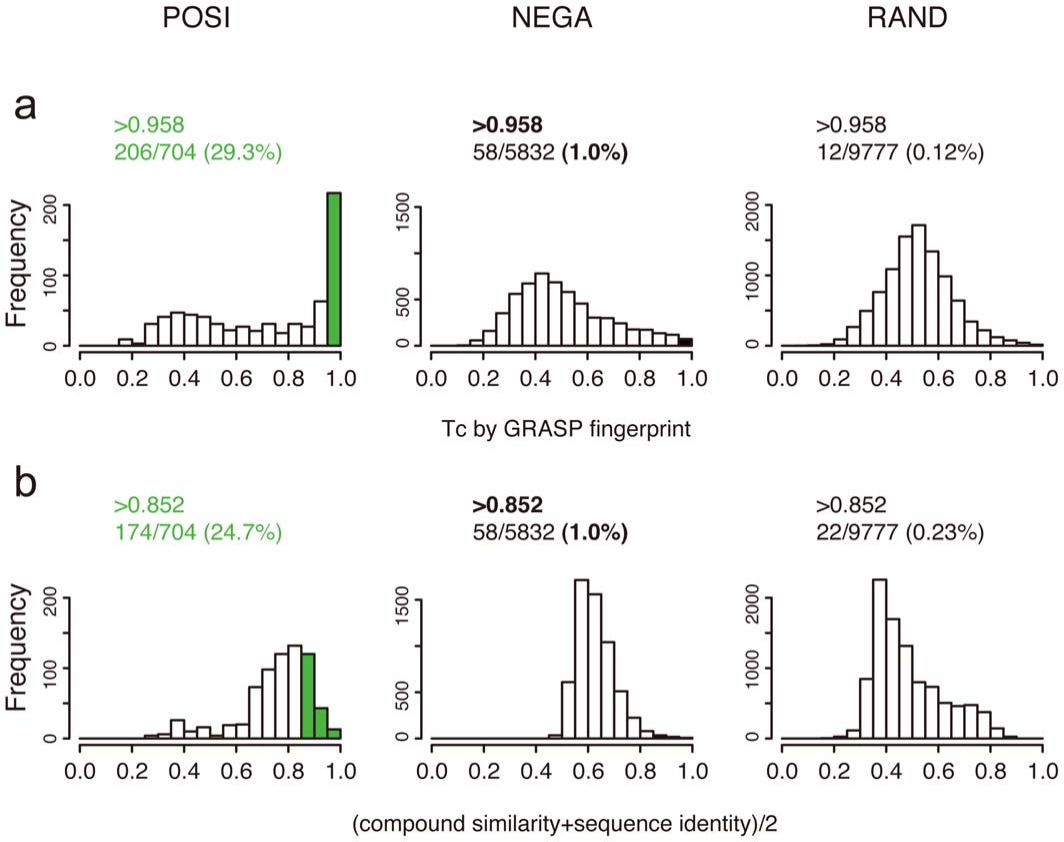
Distributions of highest scores in similarity of POSI, NEGA, and RAND to 11,219 drug-target pairs. (a) Distributions of highest Tcs of compound-protein pairs in POSI, NEGA and RAND over 11,219 drug-target pairs, in terms of GRASP fingerprints. (b) Distributions of highest similarities of compound-protein pairs in POSI, NEGA and RAND over 11,219 drug-target pairs, where the similarity is given by compound similarity plus sequence identity. Both (a) and (b) are the results obtained by only the cases that the number of substructure pairs shared between two GRASP fingerprints is more than 100.

Significant substructure pairs were small, and the GRASP-fingerprint patterns in the current drug-target pairs might also be found in non-interacting compound-protein pairs. To assess this possibility quantitatively, we used compound-protein pairs with no known interactions in DrugBank, and compound-protein pairs from other sources: 1) POSI: 1,252 ligand-protein interactions derived from PDB (Note that POSI does not have any of 11,219 interactions from DrugBank), 2) NEGA: 10,000 pairs randomly chosen from non-interacting pairs, and 3) RAND: 10,000 pairs randomly chosen from all combinations of 140,937 bioactive compounds and 6,919 druggable proteins from PubChem and Ensembl, respectively (Methods). For each pair in these datasets, we computed Tc between the GRASP fingerprint of this pair and the GRASP fingerprint of each of 11,219 drug-target pairs and checked the highest Tc among the 11,219 pairs, to search for the most similar drug-target pair in the 11,219 pairs (Methods S1). The highest Tcs of POSI were mostly in between 0.95 and 1.0, while those of NEGA and RAND were in a wider range (Fig. 6a and Fig. S4). Under the same setting, compound similarity for drugs and sequence identity for targets, instead of GRASP fingerprints, showed broader distributions of similarities for the three datasets which were overlapped with each other (Fig. 6b and Fig. S4). From these results, significant substructure pairs were more likely to appear in POSI than in NEGA and RAND. In POSI, 63% of drug-target pairs which gave the highest Tc of larger than 0.95 were not in any of R1 to R8 (Table S1), implying a different property of ligand-enzyme pairs in PDB from those in drug-target network.

### Classifying all possible compound-protein pairs by significant substructure pairs

The scalability of GRASP fingerprints on finding the most similar drug-target pair to an arbitrary given compound-protein (graph-sequence) pair was examined by generating 975,243,103 compound-protein pairs (which we call MASS) from 140,937 bioactive compounds and 6,919 druggable proteins (Methods section and Methods S1). Practical computation time for detecting the closest pair for each of all 975,243,103 pairs was totally less than 100 hours, confirming the applicability of our method to a real, large-scale drug-target analysis. Around 80% of drug-target pairs which provided the highest Tcs of larger than 0.95 were in R3, i.e. the most major cluster, implying that many unknown drug-target pairs might be in MASS (Table S1).

### Concluding remarks

Fragment-based drug design (FBDD), a widely-accepted approach in drug discovery, breaks up drug leads into smaller building blocks such as functional groups or scaffolds [14]. A basis behind FBDD is “pharmacophore”, by which common atom conformations in binding sites are used for designing multi-targeted drugs [6], and several pharmacophores over different protein families are now known [15]. These facts agree with the idea that only small portions are highly conserved structurally and electronically, being vital to drug-target binding, which supports our hypothesis. Our significant substructure pairs partitioned drug-target pairs covering most of approved drugs into clusters, which were clearly separated from each other, implying that each cluster corresponds to a unique polypharmacology type. Current analysis on drug-target networks has revealed that the true drug-target network can be much denser than currently estimated [3], but it would be hard for existing techniques, such as graph clustering or approaches using either drug or target information only, to systematically identify subnetworks, which must be denser and further harder to do that with some explanation on protein sequences and chemical structures. Our exclusive clusters suggest that intra-cluster edges should be more likely generated than inter-cluster edges, providing significant substructure pairs as a basis.

## Methods

### Data from DrugBank

DrugBank [9] is the most standard dataset that covers a wide range of drug-target interactions. The ‘small molecules’ dataset of DrugBank version 2.5 as of January 29, 2009 had 4,854 compounds where 4,730 had singly connected 2D structures and 4,191 of 4,730 had at least one known target. In more detail, 1,166 were approved, 3,014 were experimental, 284 were investigational, 77 were illicit, 62 were nutraceutical, and 35 were withdrawn compounds. The 4,191 compounds were linked to 4,362 targets, resulting in 11,219 drug-target interactions we used, and non-interacting pairs were all combinations from 4,191 compounds and 4,362 targets except the 11,219 drug-target pairs (Fig. 1a). Non-interacting pairs might contain unknown drug-target pairs, which are we think statistically negligible, since non-interacting pairs are huge. In 11,219 drug-target interactions, 1,447 (34.5%) out of 4,191 drugs were promiscuous drugs, i.e. each with at least two targets, and this percentage was consistent with 35% in Paolini et al, 2006. These promiscuous drugs were involved with 8,475 interactions (75.5% of all 11,219 drug-target pairs) and 171,029 interaction pairs. 2D structures of drugs were converted into hydrogen-suppressed molecular graphs where nodes were labeled with atom types except for hydrogen atoms and edges are labeled with bond types. All targets were treated as amino acid sequences. Thus drug substructures and target substructures mean connected subgraphs and consecutive subsequences, respectively.

### The algorithm to generate significantly co-occurring substructure pairs

We developed an efficient and scalable algorithm for mining the *N* most significant substructure pairs from given drug-target pairs (Fig. 1 and Methods S1). Our algorithm has two key features: 1) Listing up all frequent substructure pairs (Fig. 1b): This is a mathematical issue of enumerating all frequent pairs of subgraphs and subsequences which appeared in more than a pre-specified percentage (which is called *support*) in given graph-sequence pairs. Our algorithm is most efficient and scalable for this problem setting in terms of the current literature of frequent pattern mining [16]. 2) Testing significance on frequent substructure pairs (Fig. 1c): This test was performed because 1) frequent substructure pairs in drug-target pairs may frequently appear in non-interacting pairs and in this case they cannot be significant and 2) if any substructure of a substructure pair is already frequently observed in drug-target pairs rather than in non-interacting pairs, this substructure pair might not be significant even if it appears more frequently in drug-target pairs than non-interacting pairs. Thus we used a statistical test which can measure the interdependence of two substructures of a given substructure pair. This test is the same as that for detecting ‘epistasis’ in genetics [17], and we used a standard statistical test for this issue, i.e. likelihood ratio test with logistic regression (The next paragraph for detail). In reality, the two key features of our algorithm are merged into one procedure in which each time a frequent substructure pair was found, its statistical significance was tested, and if its *p*-value was lower than that of the *N*-th pair in the *N* pairs kept in our procedure, we stored the frequent substructure pair with its *p*-value and sorted the currently stored all frequent substructure pairs again according to their *p*-values; otherwise it was discarded. Practically, we obtained 41,543,488 frequent substructure pairs under the support of 5%, and *N* was 10,000. In our experiments, we used servers with Dual-Core AMD Opteron Processor 2222SE (×2) and 18G memory. Real computation time of this algorithm was around 35 hours to have the most significant 10,000 substructure pairs.

### Likelihood ratio test with logistic regression

Given drug substructure *G* and target substructure *S*, a pair of drug and target can have (1) both *G* and *S*, (2) *G* but not *S*, (3) *S* but not *G*, or (4) neither *G* nor *S*. We can use regression analysis to check the significance in the interaction between *G* and *S*. We let *X_1_* and *X_2_* be dummy variables, corresponding to a drug and a target, each of which takes 0 or 1 indicating that a drug (target) has *G* (*S*). Let *Y* be the response variable, and we pose two logistic regression models to explain *Y* (*Y = 1* for drug-target pairs and *Y = 0* for non-interacting pairs,): Letting *η = β_0_+β_1_ X_1_+β_2_ X_2_*, the probability of *Y = 1* is modeled as
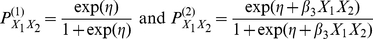



Given drug-target pairs and non-interacting pairs, these two models are fitted by maximum likelihood estimation (for which we used the Newton-Raphson method practically) independently. Then, we have two maximum likelihoods 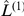 for 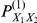, and 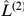 for 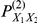. To statistically evaluate whether or not *β_3_ = 0*, that is, to evaluate whether or not there is an interaction effect between given *G* and *S*, we compare 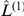 and 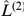 by likelihood ratio test. The test statistic 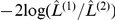 follows the chi-squared distribution with one degree of freedom under the hypothesis that *β_3_ = 0*. Thus, we can compute the *p*-value of the observed statistic, which measures the strength of joint (synergistic) effect between substructure *G* and substructure *S* in distinguishing drug-target pairs from non-interacting pairs.

### Clustering drug-target interactions through significant substructure pairs

We generated a binary matrix of taking drug-target pairs on one dimension and significant substructure pairs on the other, where if a significant substructure pair is in a drug-target pair, the corresponding element is 1; otherwise zero. On each dimension, we performed hierarchical clustering with complete linkage by using Tanimoto coefficient (Tc, which takes a value between zero and one) as the similarity of each pair of binary vectors, resulting in a dendrogram in which leave (external node) neighbors are reordered so that the nearest neighbor should be closest in terms of binary vectors. We used hierarchical clustering by the following three reasons: 1) One necessary input of partitional clustering is the number of clusters which is hard to decide while hierarchical clustering does not need the number of clusters as its input. 2) Hierarchical clustering can provide the detail of subclusters in the resultant dendrogram. 2) Hierarchical clustering is well accepted in the literature of biology and chemistry. On significant substructure pairs, we placed a cut-off value at 0.92 against 1- Tc in the dendrogram, resulting in 36 clusters. We then selected 11 largest clusters, which had more than 200 substructure pairs per cluster. These 11 clusters were manually summarized into 7 groups (G1 to G7) based on cluster similarity. On drug-target pairs, we placed a cut-off value of 0.95 against 1-Tc in the dendrogram, resulting in 279 clusters. Out of 279 clusters, we chose 13 largest clusters, which had more than 100 drug-target pairs per cluster. We further checked the cluster density, i.e. the average number of ones in all binary vectors in each cluster, and selected the eight largest clusters (R1 to R8) in terms of the cluster density. R1 to R8 covered 4,581 (40.83% of all 11,219; Table S1) rows, 3,736 (44.1%) out of all 8,475 drug-target interactions of promiscuous drugs and 47,552 out of all 171,029 interaction pairs for promiscuous drugs. We assigned the most appropriate GO term to each of R1 to R8 by casting all genes in each corresponding cluster to GoStat [18], which performs Fisher's exact test by treating the casted genes as positives (case) and all targets (proteins) in 11,219 drug-target pairs as negatives (control). For R3 which are related with G2 to G6, we checked genes which are in drug-target pairs that contain more than 80% of significant substructure pairs in each of G2 to G6, and casted these genes to GoStat to assign the most relevant GO term to each of R3-G2 to R3-G6, respectively.

### Random clusters for checking the properties of R1 to R8

A random cluster was generated by randomly selecting drug-target pairs out of the original 11,219 drug-target pairs, keeping the selected number the same as that of the corresponding cluster, i.e. one of R1 to R8. Random clusters were generated 10^5^ times, and the results were averaged over the 10^5^ runs.

### 2D-embedding of drug-target networks

We used Pajek [19] to draw the networks shown in Fig. 4. This software implements the Fruchterman-Reingold layout algorithm [20] for optimizing the network layout.

### Similarity measures used between drug-target interactions

Throughout the work, sequence identity between two targets (or proteins) was the identity of Smith-Waterman alignment obtained by using ssearch [21] (we put %identity = 0 for E>10^−10^), and compound similarity of two drugs (or compounds) was Tc between two fingerprints, each with 881 bits, given in PubChem. The compound similarity plus sequence identity was the sum of the compound similarity and the sequence identity and dividing it by 2 to have the range of this value from zero to 1.

### Datasets to confirm that significant substructure pairs cannot be found in random compound-protein pairs

In PDBbind [22], 1,300 protein-ligand structures are carefully selected from PDB as ‘refined set (version 2007)’, which is designed to be a high-quality standard dataset for theoretical studies on protein-ligand binding. However, in 1,274 cases of the 1,300 structures, the 3D structure of a protein is obtained for only a part of the entire protein, meaning that only a part of the entire sequences is kept in PDB (and PDBbind). We then manually checked the Uniprot ID of each protein of 1,274 cases and retrieved Uniprot sequences. We further removed 22 pairs, which were in our dataset of drug-target pairs, and finally obtained 1,252 protein-ligand pairs, turning into POSI. Bioactive compounds were 140,937 singly-connected components, being derived from 151,535 compounds that were marked as ‘active in any PubChem BioAssay’ in PubChem as of January 29, 2009. Druggable proteins were 6,919 proteins in Ensembl that had at least one of 191 Pfam domains specified by [23], following the definition of druggable genome [24]. RAND had 10,000 pairs randomly chosen from all combinations of 140,937 bioactive compounds and 6,919 druggable proteins.

### Scalability test

When we compute GRASP fingerprints of all 975,143,103 combinations of 140,937 bioactive compounds and 6,919 druggable proteins, we divided these pairs into 16 blocks of an equal size and assigned each block to a CPU. Practical computation time to obtain 1,000,000 pairs with Tc of not less than 0.9 was around 90–98 hours for a block.

## Supporting Information

Figure S1 An enlargement of the matrix of Fig. 3, including all major clusters of both significant substructure pairs and drug-target pairs. From left to right on the three columns on the right-hand side of the matrix, in the first column, an element is colored red if the corresponding drug-target pair has an approved drug; otherwise blue. In the middle column, an element is colored red if the corresponding drug-target pair has GPCR; otherwise blue. In the right column, an element colored red if the target of the corresponding drug-target pair is a membrane protein; otherwise blue.
(PDF)

Figure S2 Hierarchical clustering on drug-target pairs where R1 to R8 are specified with brief descriptions on representative drug-target pairs or targets.
(PDF)

Figure S3 Symmetric heatmaps of similarities among 11,219 drug-target pairs in terms of (a) GRASP fingerprints, (b-1) compound similarity plus sequence identity, (b-2) compound similarity only and (b-3) sequence identity only, where a brighter dot shows a higher similarity. In (a) the order of drug-target pairs in rows follows that of Fig. 2 of the main text. In (b-1), the order of drug-target pairs in rows is arranged so that the most similar pair in terms of compound similarity plus sequence identity should be as nearest as possible. In (b-2) and (b-3), the order of drug-target pairs in rows follows that of (b-1).
(PDF)

Figure S4 (a) Distributions of highest Tc of compound-protein pairs in POSI, NEGA and RAND, when the Tc was computed between the GRASP fingerprints of two pairs. (b) Distributions of highest similarities of compound-protein pairs in POSI, NEGA and RAND, when the similarity is given by compound similarity plus sequence identity.
(PDF)

Methods S1 Full details of the proposed algorithms.
(PDF)

Table S1 For each of R1 to R8, (a) the number of drug-target pairs, (b) the number of drug-target pairs which gave the highest Tc of larger than 0.95 for pairs in POSI and (c) the number of drug-target pairs which gave the highest Tc of larger than 0.95 for pairs in MASS.
(PDF)

Table S2 The number of drug-target pairs with the number of drugs and targets in each of R1 to R8.
(PDF)

Table S3 The most related 30 GO terms with each of R1 to R8. The most right column shows the value of the number of genes in both the corresponding cluster and the corresponding GO term divided by the number of genes in the corresponding GO term.
(PDF)

Table S4 For each of R1 to R8, the average Tc of paired GRASP fingerprints over all drug-target pairs in the corresponding cluster, and that over 105 clusters, each having interactions randomly selected out of the original 11,219 drug-target pairs and keeping the cluster size the same as that of the corresponding cluster.
(PDF)

Table S5 For each of R1 to R8, the average Tc of paired GRASP fingerprints over drug-target pairs of all promiscuous drugs in the corresponding cluster, and that over 105 clusters, each having interactions randomly selected out of the original 8,475 promiscuous drug-target pairs and keeping the cluster size the same as that of the corresponding cluster.
(PDF)

Table S6 In each of R1 to R8, given drugs and targets of drug-target pairs, the ratio of drug-target pairs which were in the corresponding cluster to all drug-target pairs between them, and the average over those of 105 clusters, each having drug-target pairs randomly selected out of the original 11,219 drug-target interactions and keeping the cluster size the same as that of the corresponding cluster.
(PDF)

Table S7 In each of R1 to R8, given drugs and targets of drug-target pairs (of promiscuous drugs), the ratio of drug-target pairs which were in the corresponding cluster to all drug-target pairs between them, and the average over those of 105 clusters, each having drug-target pairs (of promiscuous drugs) randomly selected out of the original 11,219 drug-target interactions and keeping the cluster size the same as that of the corresponding cluster.
(PDF)

Table S8 The number of drug-target pairs sharing the same drugs and the average sequence identity between target (amino acid sequences) of these pairs.
(PDF)
