# Supplementary material for: Mining Significant Substructure Pairs for Interpreting Polypharmacology in Drug-Target Network
Source: PLoS One. 2011 Feb 23;6(2):e16999. doi: 10.1371/journal.pone.0016999 (PMC3044142; doi:10.1371/journal.pone.0016999)
Supplement: Figure S1 — An enlargement of the matrix of Fig. 3, including all major clusters of both significant substructure pairs and drug-target pairs. From left to right on the three columns on the right-hand side of the matrix, in the first column, an element is colored red if the corresponding drug-target pair has an approved drug; otherwise blue. In the middle column, an element is colored red if the corresponding drug-target pair has GPCR; otherwise blue. In the right column, an element colored red if the target of the corresponding drug-target pair is a membrane protein; otherwise blue. (PDF) [file pone.0016999.s001.pdf]

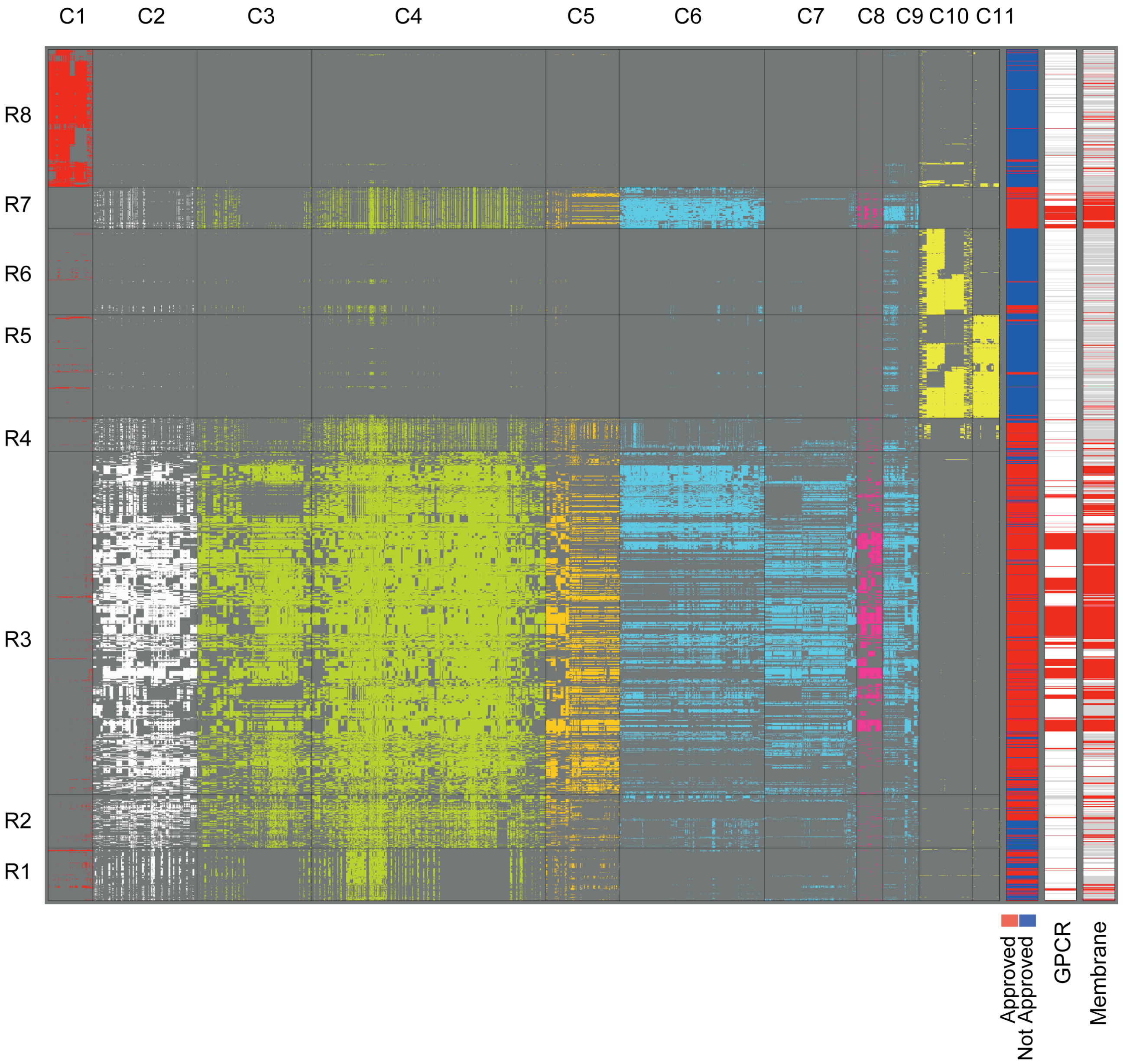

Figure S1: An enlargement of the matrix of Fig.3, including all major clusters of both significant substructure pairs and drug-target pairs. From left to right on the three columns on the right-hand side of the matrix, in the first column, an element is colored red if the corresponding drug-target pair has an approved drug; otherwise blue. In the middle column, an element is colored red if the corresponding drug-target pair has GPCR; otherwise blue. In the right column, an element colored red if the target of the corresponding drug-target pair is a membrane protein; otherwise blue.
