# Supplementary material for: Mining Significant Substructure Pairs for Interpreting Polypharmacology in Drug-Target Network
Source: PLoS One. 2011 Feb 23;6(2):e16999. doi: 10.1371/journal.pone.0016999 (PMC3044142; doi:10.1371/journal.pone.0016999)
Supplement: Figure S2 — Hierarchical clustering on drug-target pairs where R1 to R8 are specified with brief descriptions on representative drug-target pairs or targets. (PDF) [file pone.0016999.s002.pdf]

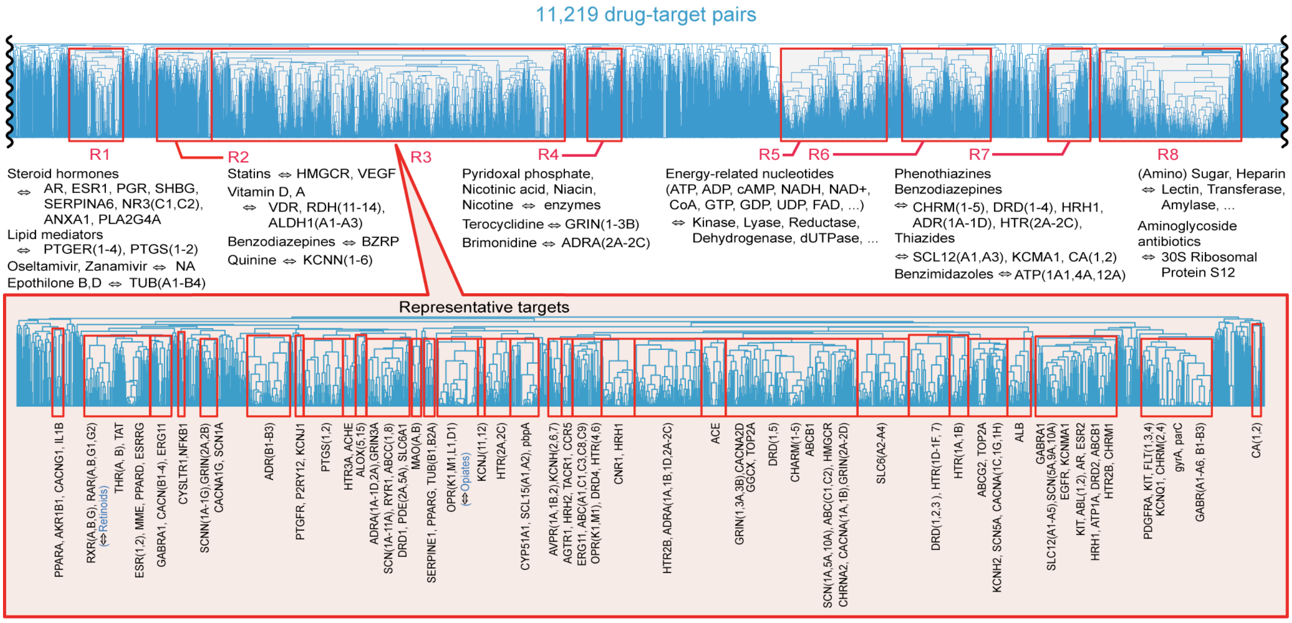

Figure S2: Hierarchical clustering on drug-target pairs where R1 to R8 are specified with brief descriptions on representative drug-target pairs or targets.
