# Supplementary material for: Mining Significant Substructure Pairs for Interpreting Polypharmacology in Drug-Target Network
Source: PLoS One. 2011 Feb 23;6(2):e16999. doi: 10.1371/journal.pone.0016999 (PMC3044142; doi:10.1371/journal.pone.0016999)
Supplement: Figure S3 — Symmetric heatmaps of similarities among 11,219 drug-target pairs in terms of (a) GRASP fingerprints, (b-1) compound similarity plus sequence identity, (b-2) compound similarity only and (b-3) sequence identity only, where a brighter dot shows a higher similarity. In (a) the order of drug-target pairs in rows follows that of Fig. 2 of the main text. In (b-1), the order of drug-target pairs in rows is arranged so that the most similar pair in terms of compound similarity plus sequence identity should be as nearest as possible. In (b-2) and (b-3), the order of drug-target pairs in rows follows that of (b-1). (PDF) [file pone.0016999.s003.pdf]

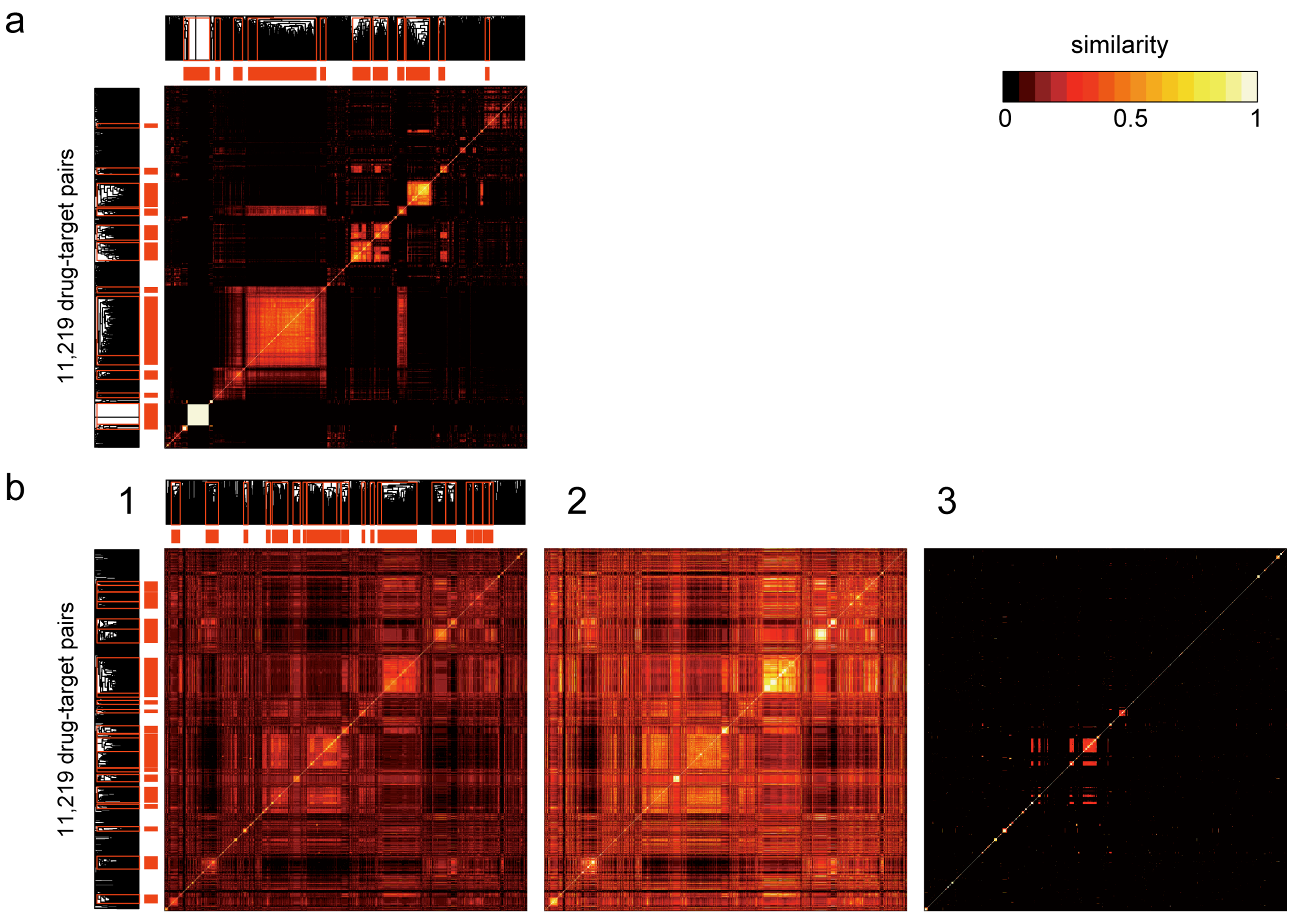

Figure S3: Symmetric heatmaps of similarities among 11,219 drug-target pairs in terms of (a) GRASP fingerprints, (b-1) compound similarity plus sequence identity, (b-2) compound similarity only and (b-3) sequence identity only, where a brighter dot shows a higher similarity. In (a) the order of drug-target pairs in rows follows that of Fig. 2 of the main text. In (b-1), the order of drug-target pairs in rows is arranged so that the most similar pair in terms of compound similarity plus sequence identity should be as nearest as possible. In (b-2) and (b-3), the order of drug-target pairs in rows follows that of (b-1).
