# Supplementary material for: Mining Significant Substructure Pairs for Interpreting Polypharmacology in Drug-Target Network
Source: PLoS One. 2011 Feb 23;6(2):e16999. doi: 10.1371/journal.pone.0016999 (PMC3044142; doi:10.1371/journal.pone.0016999)
Supplement: Figure S4 — (a) Distributions of highest Tc of compound-protein pairs in POSI, NEGA and RAND, when the Tc was computed between the GRASP fingerprints of two pairs. (b) Distributions of highest similarities of compound-protein pairs in POSI, NEGA and RAND, when the similarity is given by compound similarity plus sequence identity. (PDF) [file pone.0016999.s004.pdf]

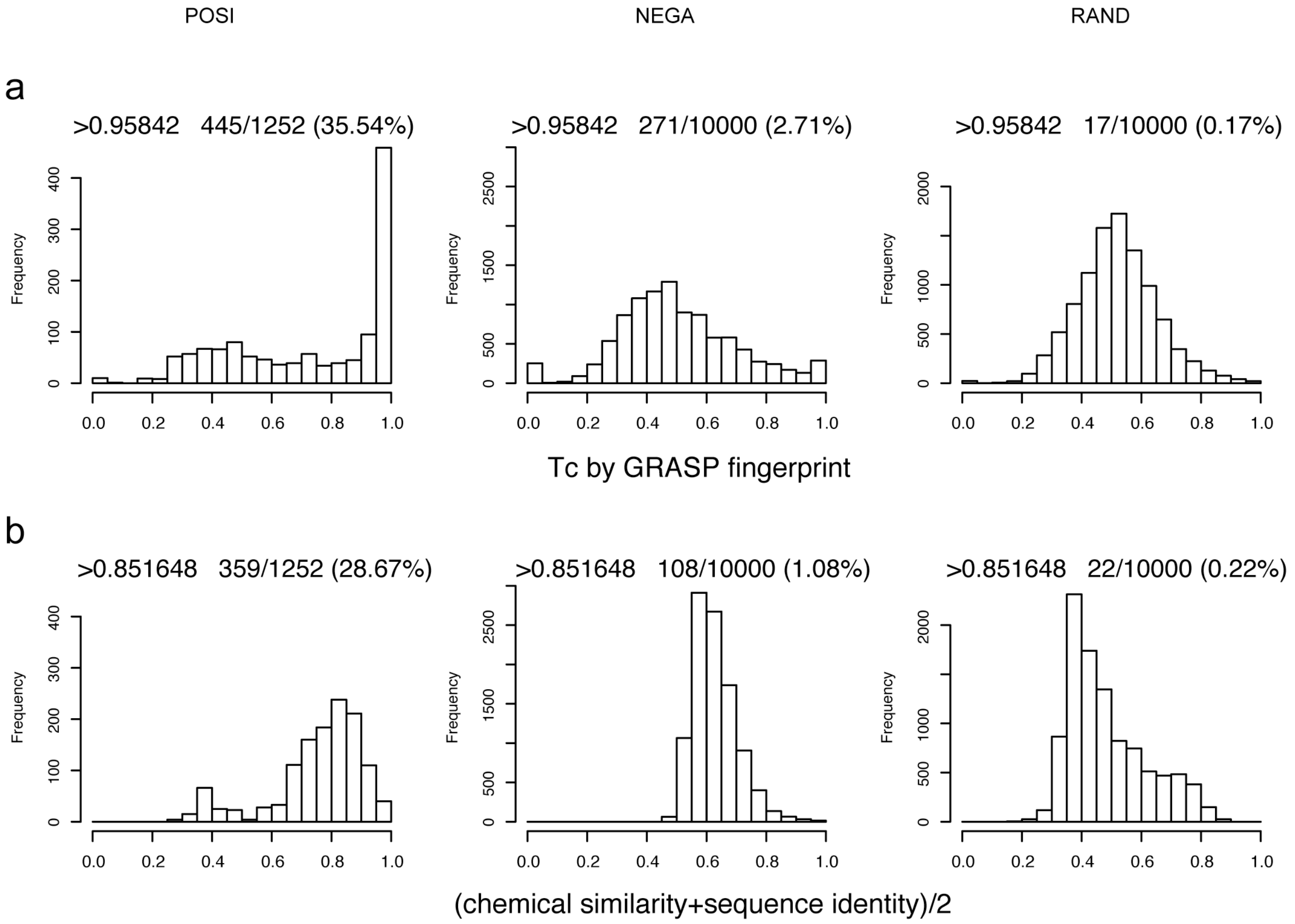

Figure S4: (a) Distributions of highest Tc of compound-protein pairs in POSI, NEGA and RAND, when the Tc was computed between the GRASP fingerprints of two pairs. (b) Distributions of highest similarities of compound-protein pairs in POSI, NEGA and RAND, when the similarity is given by compound similarity plus sequence identity.
