# Supplementary material for: Mining Significant Substructure Pairs for Interpreting Polypharmacology in Drug-Target Network
Source: PLoS One. 2011 Feb 23;6(2):e16999. doi: 10.1371/journal.pone.0016999 (PMC3044142; doi:10.1371/journal.pone.0016999)
Supplement: Methods S1 — Full details of the proposed algorithms. (PDF) [file pone.0016999.s005.pdf]

Supplementary Methods for 'Mining significant substructure pairs for interpreting polypharmacology in drug-target network'

Ichigaku Takigawa, Koji Tsuda and Hiroshi Mamitsuka

This supplement provides detailed description on computational methods used in the work, consisting of mainly four parts: 1) mining frequent subgraph-subsequence pairs (Section 1), 2) evaluating the significance of generated substructure pairs (Section 2), 3) generating the GRASP fingerprint of an arbitrary given compound-protein pair (Section 3), and 4) comparing two compound-protein pairs in terms of GRASP fingerprints (Section 4).

1 Mining frequent subgraph-subsequence pairs

We represent drug-target (or compound-protein) pairs by graph-sequence pairs. An essential task in the work is to find subgraph-subsequence pairs, which significantly occur in drug-target pairs, comparing to non-interacting pairs. For this problem, our algorithm has two key steps: (1) We enumerate all subgraph-subsequence pairs frequently occurring in drug-target pairs, and (2) we evaluate the significance of frequent subgraph-subsequence pairs. We describe (1) in this section, while (2) is described in the next section.

1.1 Mining frequent patterns

We start with a general but intuitive explanation on terms used throughout this document. A database is a set of instances, such as itemsets, sequences, trees and graphs. Given a database, say a database on graphs, the *support* of a subgraph is the number of graphs in the database having this subgraph. A *frequent* pattern is a subgraph, which occurs in at least σ graphs for a given threshold σ, which we call the *minimum support.* That is, the support of a frequent subgraph is larger than or equal to the minimum support. In the work, we focus on graph-sequence pairs and here give notations for graph-sequence pairs.

Definition 1 (Support)**.** Let $\mathcal{G}$ and $\mathcal{S}$ be a set of graphs and a set of sequences, respectively. For subgraph g, the support of g in $\mathcal{G}$, denoted by $\mathrm{support}(g|\mathcal{G})$, is defined as the number of graphs in $\mathcal{G}$ containing g. Similarly, for subsequence s, the support of s in $\mathcal{S}$, denoted by $\mathrm{support}(s|\mathcal{S})$, is defined as the number of sequences in $\mathcal{S}$ containing s.

Definition 2 (Instances and marginal support)**.** Let $\mathcal{Q}$ be a set of graph-sequence pairs. For a subgraph-subsequence pair (g, s), the pairs in $\mathcal{Q}$ containing (g, s) are called the instances of (g, s) in Q, denoted by $Q_{(g,s)} := \{(G, S) \in \mathcal{Q} \mid g \subseteq G, s \subseteq S\}$. The support of (g, s) in $\mathcal{Q}$, denoted by $\mathrm{support}((g, s)|\mathcal{Q})$, is defined as the size of instances in $\mathcal{Q}$ containing (g, s)

$$\mathrm{support}((g, s)|\mathcal{Q}) := |\mathcal{Q}_{(g,s)}| = |\{(G, S) \in \mathcal{Q} \mid g \subseteq G, s \subseteq S\}|.$$

The marginal support of g in $\mathcal{Q}$, denoted by $\mathrm{support}((g, *)|\mathcal{Q})$, is defined as $|\{(G, S) \in \mathcal{Q} \mid g \in G\}|$, and that of s in $\mathcal{Q}$, denoted by $\mathrm{support}((*, s)|\mathcal{Q})$, is defined by $|\{(G, S) \in \mathcal{Q} \mid s \in S\}|$.

Definition 3. (Bipartite-graph representation and marginal sets) A set $\mathcal{Q}$ of graph-sequence pairs can be represented as a bipartite graph $(\mathcal{Q}, \mathcal{G}, \mathcal{S})$ with two node sets $\mathcal{G}$ and $\mathcal{S}$ and edge set $\mathcal{Q}$. The two sets $\mathcal{G}$ and $\mathcal{S}$ can be determined directly from $\mathcal{Q}$ as the minimum sets $\mathcal{G}_\mathcal{Q} := \{G \mid (G, S) \in \mathcal{Q}\}$ and $\mathcal{S}_\mathcal{Q} := \{S \mid (G, S) \in \mathcal{Q}\}$. We call $\mathcal{G}_\mathcal{Q}$ and $\mathcal{S}_\mathcal{Q}$ marginal sets from $\mathcal{Q}$.

Remark 1. Note that $\mathrm{support}(g|\mathcal{G}_\mathcal{Q})$ and $\mathrm{support}((g, *)|\mathcal{Q})$ are different quantities since G can contain g multiple times in $\mathcal{Q}$. We can compute $\mathrm{support}(g|\mathcal{G}_\mathcal{Q})$ from graph set $\mathcal{G}_\mathcal{Q}$ only, whereas $\mathrm{support}((g, *)|\mathcal{Q})$ requires pair set $\mathcal{Q}$. The same holds true for $\mathrm{support}(s|\mathcal{S}_\mathcal{Q})$ and $\mathrm{support}((*, s)|\mathcal{Q})$.

Remark 2. In bipartite graph $(\mathcal{Q}, \mathcal{G}_\mathcal{Q}, \mathcal{S}_\mathcal{Q})$, marginal support $\mathrm{support}((g, *)|\mathcal{Q})$ equals to the sum of degrees of nodes $\{G \in \mathcal{G}_\mathcal{Q} | g \subseteq G\}$. Similarly marginal support $\mathrm{support}((*, s)|\mathcal{Q})$ equals to the sum of degrees of nodes $\{S \in \mathcal{S}_\mathcal{Q} | s \subseteq S\}$.

Definition 4 (Frequent subgraph-subsequence pairs)**.** Given minimum support σ and a set of graph-sequence pairs $\mathcal{Q}$, frequent subgraph-subsequence pairs $\mathcal{F}$ are all subgraph-subsequence pairs, each with a support which is not less than σ, that is, formally defined by $\mathcal{F} := \{(g, s) \mid \text{support}((g, s)|\mathcal{Q}) \geqslant \sigma\}$. A subgraph-subsequence pair, which is not in $\mathcal{F}$, is infrequent.

1.2 Pattern-growth approach for mining frequent patterns

If a trivial, brute-force approach is taken for mining frequent patterns, a serious problem is efficiency, and so we need to develop a time-efficient algorithm. Key factors for efficiency are (1) optimizing the order of patterns to be explored, (2) minimizing the time interval from one pattern to the next, and (3) eliminating redundant search without overlooking frequent patterns. One important point to consider these factors is that patterns, such as subgraphs and subsequences, often contain smaller patterns, which are simply redundant. This inclusive relation, which can be summarized into an idea called "downward closure" property, can be considered to make a brute-force algorithm efficient.

Proposition 1 (Downward closure)**.** *If a pattern P contains any smaller infrequent patterns, then P is infrequent.*

This property naturally leads to a so-called *pattern-growth* approach: we start with smallest patterns and extend to larger patterns, and during this process if we find an infrequent pattern, then we do not need to extend it more because of the downward closure property. More concretely, for graphs, we start with all possible graphs with one edge and for each of them, you do not have to extend it if it is an infrequent subgraph; otherwise you can add an edge to extend. This pattern growth procedure forms a hierarchy, which can be represented in a rooted ordered tree (Fig. 1b), called an 'enumeration tree'. The null pattern is on the root, subgraphs with only one edge are on the children of the root. A subgraph with a larger number of edges are on the nodes in a deeper level. Note that the support of a subgraph is monotonically decreasing with increasing the size of the subgraph, meaning a subgraph on a deeper level with a smaller support. The enumeration tree is a spanning tree on all frequent subgraphs, each node corresponding to a frequent subgraph in a one-to-one manner. By traversing this tree as a search space, all subgraphs can be enumerated completely without any duplication. This tree-shaped search space thus ensures the uniqueness and completeness on searching frequent subgraphs. This can be applied to other frequent patterns, say subsequences, for which an enumeration tree can be represented as in Fig. 1a.

Proposition 2 (Enumeration tree)**.** *A pattern-growth approach, often implicitly, produces a rooted ordered spanning tree on frequent patterns to be enumerated. This tree is called an enumeration tree.*

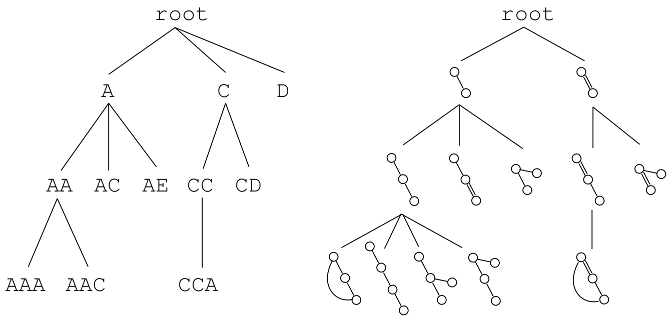

Figure 1: Samples of enumeration trees for (a) frequent subsequences and for (b) frequent subgraphs.

Because of the nature of a tree, a node in an enumeration tree has only one parent node, which has more than one child nodes. A pattern on a child is a larger but minimum pattern. For example, AC in Fig. 1 (a) is a longer but minimum of A. Note that in this sense, AC can be a child of C but is exactly not a child of C in this figure. The unique parent-child relationship in an enumeration tree depends on an individual mining algorithm, in which some order of sibling nodes is often simply used. In Fig. 1 (a), A is prior to C, turning into that AC from A is prior to AC from C.

In recent years, enumeration algorithms based on the depth-first search by an enumeration tree have been attracting attention due to an advantage in computational efficiency. We then used depth-first search-based algorithms for frequent pattern mining. That is, we used the PrefixSpan algorithm [1] for building an enumeration tree of frequent subsequences and the gSpan algorithm [2] for frequent subgraphs. Note that the original PrefixSpan algorithm allows any size of gaps in subsequences, but we restrict to only consecutive subsequences. This is because input sequences are amino acid sequences, which are usually long and consist of only twenty amino acids, meaning that if we allow any size of gaps, small subsequences are likely to be frequent, by which mining subsequences in protein sequences will be infeasible in our case.

1.3 Mining frequent subgraph-subsequence pairs

The algorithm in the work solves the problem of mining frequent subgraph-subsequence pairs by implicitly combining two enumeration trees, one for frequent subgraphs and the other for frequent subsequences. First, we consider all combinations

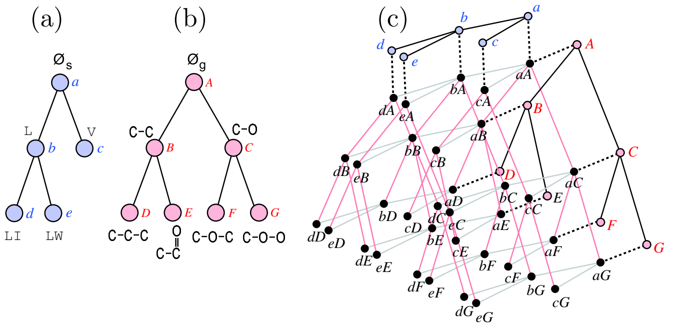

Figure 2: An example of the search space used in the algorithm. The search space (c) is defined as the graph product of two enumeration trees for subsequences (a) and for subgraphs (b). It covers all possible pairs of frequent subgraphs and subsequences.

of frequent subgraphs and subsequences which can cover all frequent subgraph-subsequence pairs from the definition. The search space for all these combinations can be defined by a product graph of two enumeration trees. For example, we can define the search space of all subgraph-subsequence combinations by two enumeration trees first, shown in Fig. 2a and Fig. 2b, and they can be combined into Fig. 2c, where each subgraph-subsequence pair has two parent nodes, and hence this is no longer a tree. In this case, theoretically, we can compute the support of each subgraph-subsequence pair in a dynamic-programming manner.

Definition 5. (Search space for subgraph-subsequence pairs) Let $\mathcal{T}_g$ and $\mathcal{T}_s$ be enumeration trees for subgraphs and subsequences, respectively. Then all frequent subgraph-subsequence pairs $\mathcal{F}$ in Definition 4 are included in $\mathcal{T}_g \times \mathcal{T}_s$, where $\times$ indicates a graph product.

Proposition 3 (Dynamic programming for subgraph-subsequence pairs)**.** *Let $\mathcal{T}_g$ and $\mathcal{T}_s$ be enumeration trees for frequent subgraphs and subsequences, respectively. Theoretically, we can compute all instances of (g, s) in $\mathcal{Q}$, that is, $\mathcal{Q}_{(g,s)}$, for any frequent g and s in the following recursions:*

1. $\mathcal{Q}_{(g,s)} = \mathcal{Q}_{(\mathrm{parent}(g),s)} \cap \mathcal{Q}_{(g,\mathrm{parent}(s))}$.
2. $\mathcal{Q}_{(g,\varnothing_s)} = \{(G, S) \in \mathcal{Q} \mid g \in G\}$.
3. $\mathcal{Q}_{(\varnothing_g,s)} = \{(G, S) \in \mathcal{Q} \mid s \in S\}$.

where $\mathrm{parent}(g)$ *represents a subgraph of the parent of a node to which g is assigned in $\mathcal{T}_g$, and* $\mathrm{parent}(s)$ *represents a subsequence of the parent of a node to which s is assigned in $\mathcal{T}_s$. The $\varnothing_g$ and $\varnothing_s$ indicates the root nodes of $\mathcal{T}_g$ and $\mathcal{T}_s$, respectively.*

In practice, all combinations of frequent subgraphs and subsequences can include many infrequent subgraph-subsequence pairs, and we can also use the downward closure property on the product graph of two enumeration trees to eliminate redundancy in enumeration. For example, if (L,C-C) at node bB in Fig. 2c is infrequent, patterns at nodes bD, bE, dB, eB, dD, eD, dE and eE must be all infrequent.

Proposition 4 (Two-way downward closure for subgraph-subsequence pairs)**.** *If a subgraph-subsequence pair (g, s) is infrequent, then all subgraph-subsequence pairs (g', s') (where $g \subseteq g'$ and $s \subseteq s'$) are infrequent. This means that if* $\mathrm{support}((g, s)|\mathcal{Q}) = |\mathcal{Q}_{(g,s)}| < \sigma$*, then there is no need to extend (g, s) further.*

If the recursion rules in Proposition 3 are used directly, all instances explicitly need to be kept in the graph product $\mathcal{T}_g \times \mathcal{T}_s$. That is, at each node of pattern (g, s), instances $\mathcal{Q}_{(g,s)}$ (which should be passed to subsequent nodes) must be kept. This manner explicitly tends to result in a time- and space-consuming procedure, because two enumeration trees are practically very huge. Thus our implementation passes these instances implicitly along with a depth-first traversal of the graph product $\mathcal{T}_g \times \mathcal{T}_s$ by simplifying recursion rules into those in Proposition 5. This depth-first traversal, which is similar to the gSpan and PrefixSpan algorithms, gives a practically efficient algorithm.

Proposition 5 (A simplified recursion rule)**.** *The recursion rules in Proposition 3 can be simplified to*

$$\mathcal{Q}_{(g,s)} = \mathcal{Q}_{(\mathrm{parent}(g),s)} \cap \{(G, S) \in \mathcal{Q} \mid g \in G\} \quad \textit{and} \quad \mathcal{Q}_{(\varnothing_g,s)} = \{(G, S) \in \mathcal{Q} \mid s \in S\}.$$

Proof. From $\mathcal{Q}_{(g,s)} = \mathcal{Q}_{(\mathrm{parent}(g),s)} \cap \mathcal{Q}_{(g,\mathrm{parent}(s))}$ and $\mathcal{Q}_{(g,\mathrm{parent}(s))} \subseteq \mathcal{Q}_{(\mathrm{parent}(g),\mathrm{parent}(s))}$, we have

$$\mathcal{Q}_{(g,s)} = \mathcal{Q}_{(\mathrm{parent}(g),s)} \cap \mathcal{Q}_{(\mathrm{parent}(g),\mathrm{parent}(s))} \cap \mathcal{Q}_{(g,\mathrm{parent}(\mathrm{parent}(s)))} = \mathcal{Q}_{(\mathrm{parent}(g),s)} \cap \mathcal{Q}_{(g,\mathrm{parent}(\mathrm{parent}(s)))}.$$

By repeatedly applying this equation, we have

$$\mathcal{Q}_{(g,s)} = \mathcal{Q}_{(\text{parent}(g),s)} \cap \mathcal{Q}_{(g,\text{parent}(\text{parent}(s)))} = \mathcal{Q}_{(\text{parent}(g),s)} \cap \mathcal{Q}_{(g,\text{parent}(\text{parent}(\text{parent}(s))))} = \cdots = \mathcal{Q}_{(\text{parent}(g),s)} \cap \mathcal{Q}_{(g,\varnothing_s)}.$$

□

We explain an efficient way to obtain an arbitrary $\mathcal{Q}_{(g,s)}$ by simplified rules in Proposition 5. The algorithm first traverses $\mathcal{T}_s$ until s is found. This is implicitly done by an algorithm for mining frequent subsequences by using marginal support $\text{support}((*,s)|\mathcal{Q})$ instead of $\text{support}(s|\mathcal{S}_\mathcal{Q})$, i.e. the support in marginal set $\mathcal{S}_\mathcal{Q}$. When s is found, we have instances $\mathcal{Q}_{(\varnothing_g,s)}$. Then, the algorithm starts to traverse $\mathcal{T}_g$ from node $(\varnothing_g, s)$, keeping instances $\mathcal{Q}_{(\cdot,s)}$. When moving on from $(\text{parent}(x), s)$ to (x, s), the algorithm can reduce instances by the first rule: $\mathcal{Q}_{(x,s)} = \mathcal{Q}_{(\text{parent}(x),s)} \cap \mathcal{Q}_{(x,\varnothing_s)}$ where the extension of $\text{parent}(x)$ to x is considered. In this way, the algorithm traces instances $\mathcal{Q}_{(g,s)}$ along with the path from $\mathcal{Q}_{(g,\varnothing_s)}$ to $\mathcal{Q}_{(g,s)}$. Note that this holds true when g is replaced with s, since g and s are symmetric in the rules in Proposition 3. Note further that this simplification works more if a larger enumeration tree is examined first. In reality, Proposition 5 examines s first since $\mathcal{T}_s$ is expected to be larger than $\mathcal{T}_g$ in drug-target pairs. Note that we can see the size of $\mathcal{T}_g$ ($\mathcal{T}_s$) by traversing $\mathcal{T}_g$ ($\mathcal{T}_s$) in terms of the marginal support in $\mathcal{Q}$ by applying an algorithm for mining frequent subgraphs (subsequences).

Proposition 6 (Procedure for exactly searching frequent subgraph-subsequence pairs)**.** *Using an algorithm for mining frequent subgraphs and that for mining frequent subsequences, we can enumerate frequent subgraph-subsequence pairs as follows.*

1. *Compute $\mathcal{Q}_{(g,\varnothing_s)}$ for all possible g using a frequent subgraph mining algorithm in terms of* $\text{support}((g,*)|\mathcal{Q})$.
   (Build enumeration tree $\mathcal{T}_g$ for efficiency)

2. *Start a frequent subsequence mining algorithm from $(\varnothing_g, \varnothing_s)$:*
   (Implicitly traverse enumeration tree $\mathcal{T}_s$ from the root)

   For each $s \in \mathcal{T}_s$ in a depth-first traversal order:

   For each $g \in \mathcal{T}_g$ in a depth-first traversal order:

   - *continue if $g = \varnothing_g$ or $s = \varnothing_s$.*
   - *reduce instances $\mathcal{Q}_{(g,s)}$ by $\mathcal{Q}_{(g,s)} = \mathcal{Q}_{(\text{parent}(g),s)} \cap \mathcal{Q}_{(g,\varnothing_s)}$.*
   - *compute* $\text{support}((g,s)|\mathcal{Q}) = |\mathcal{Q}_{(g,s)}|$.
   - *break if* $\text{support}((g,s)|\mathcal{Q}) < \sigma$.

Figure 3 shows a toy example of mining subgraph-subsequence pairs for the minimum support of 3. Assume that we have 10 graph-sequence pairs numbered as $1, 2, \cdots, 10$, each containing corresponding subgraph-subsequence pairs as indicated in Fig. 3. For example, only graph-sequence pairs 4 and 5 include (C-C, L) and only 6 and 7 include (C-O-O,LW). Parent-child relationships in two enumeration trees in Figure 2 are also indicated as lines at both sides. Our purpose is to find all frequent pairs colored in white: In this example, frequent pairs are only five: (C-O,L), (C-O,V), (C-O-C,L), (C-O-O,L), and (C-O-O,V). The procedure in Proposition 6 first builds enumeration tree $\mathcal{T}_g$, which corresponds to computing all instances in the first row of Figure 3, and then starts traversing enumeration tree $\mathcal{T}_s$ from the root. By traversing $\mathcal{T}_s$ in a depth-first manner, the first pattern to be found is (B, b). Instances containing (B, b) can be computed by the intersection between $\mathcal{Q}_{(B,a)} = \{1, 2, 3, 4, 5\}$ and $\mathcal{Q}_{(A,b)} = \{4, 5, 6, 7, 8, 10\}$, resulting in $\mathcal{Q}_{(B,b)} = \{4, 5\}$. Since (B, b) is infrequent (i.e. $|\mathcal{Q}_{(B,b)}| < 3$), we do not have to proceed to subsequent (D, b) and (E, b). Then, the next pattern to be found is (C, b), which turns out to be frequent. We then move on to (F, b), since $\text{parent}(F) = C$. Instances $\mathcal{Q}_{(F,b)}$ is computed by $\mathcal{Q}_{(F,b)} = \mathcal{Q}_{(C,b)} \cap \mathcal{Q}_{(F,a)} = \{4, 5, 6\}$. Similarly, we can compute the instances of the next pattern (G, b) by $\mathcal{Q}_{(G,b)} = \mathcal{Q}_{(C,b)} \cap \mathcal{Q}_{(G,a)} = \{6, 7, 8\}$. Since we traverse all nodes of $\mathcal{T}_g$ for $b \in \mathcal{T}_s$ at this moment, then we proceed to the next pattern (B, d) by traversing $\mathcal{T}_s$ from b to d. However, since subsequent nodes, (B, d), (C, d), (B, e), (C, e), (B, c), are all infrequent, then the next frequent pattern to be found is (C, c). Then the subsequent (F, c) and (G, c) are evaluated in this order, and we found that (F, c) is infrequent but (G, c) is frequent. Then, we have no nodes to proceed in $\mathcal{T}_g \times \mathcal{T}_s$, and the procedure is terminated. Finally we obtain all five frequent patterns (C, b), (F, b), (G, b), (C, c), and (G, c) in this order.

2 Evaluating significance of subgraph-subsequence pairs

The goal of the algorithm in the work has two steps: (1) Enumerating all subgraph-subsequence pairs frequently occurring in drug-target pairs. (2) Evaluating the significance of each frequent substructure pair. We describe (2) in this section.

More concretely, the statistical test we used in the work is the same as that for detecting 'epistasis' in genetics [3]. We will describe this test as well as an efficient implementation of this test in the subsequent sections, starting with likelihood ratio test with logistic regression.

		A	B	C	D	E	F	G
		$\emptyset_g$	C-C	C-O	C-C-C	C-C(=O)	C-O-C	C-O-O
a	$\emptyset_s$	1 2 3 4 5 6 7 8 9 10	1 2 3 4 5	4 5 6 7 8 9	1 2 3	3 4 5	4 5 6	6 7 8 9
b	L	4 5 6 7 8 10	4 5	4 5 6 7 8	none	4 5	4 5 6	6 7 8
c	V	7 8 9 10	none	7 8 9	none	none	none	7 8 9
d	LI	4 5 10	4 5	4 5	none	4 5	4 5	none
e	LW	6 7 10	none	6 7	none	none	6	6 7

Figure 3: An example for searching all subgraph-subsequence pairs common in 3 or more of 10 graph-sequence pairs numbered $1, 2, \ldots, 10$. This table corresponds to the enumeration trees of Fig. 2a and Fig. 2b.

2.1 Likelihood ratio test using logistic regression

The probability p that an event occurs given d explanatory variables $x_1, x_2, \ldots, x_d$, can be shown by logistic regression in the following manner:

$$p = \text{Prob}\{\text{the event occurs} \mid x_1, x_2, \ldots, x_d\} = \frac{\exp(\eta)}{1+\exp(\eta)} = \frac{1}{1+\exp(-\eta)},$$

where $\eta = \theta_0 + \theta_1 x_1 + \cdots + \theta_d x_d$ is a (linear) composite variable. Note that p takes a value between zero and one due to the logistic function, even though η ranges from $-\infty$ to ∞. Note further that this equation can be transformed into $\log\{p/(1-p)\} = \eta$ where $\text{Prob}\{\text{the event occurs} \mid x_1, x_2, \ldots, x_d\}/\text{Prob}\{\text{the event does not occur} \mid x_1, x_2, \ldots, x_d\} = p/(1-p)$.

Let $Y \in \{0, 1\}$ be a binary response variable, where the probability of $Y = 0$ (and $Y = 1$) is modeled by

$$\text{Prob}\{Y=0\} = 1 - p_{\boldsymbol{\theta}}(\boldsymbol{X}) \quad \text{and} \quad \text{Prob}\{Y=1\} = p_{\boldsymbol{\theta}}(\boldsymbol{X}), \quad p_{\boldsymbol{\theta}}(\boldsymbol{X}) = \frac{\exp(\boldsymbol{\theta}'\boldsymbol{Z})}{1+\exp(\boldsymbol{\theta}'\boldsymbol{Z})},$$

where $\boldsymbol{\theta} = (\theta_0, \theta_1, \ldots, \theta_d)'$ and $\boldsymbol{Z} = (1, \boldsymbol{X}')' = (1, X_1, X_2, \ldots, X_d)'$. To fit this model to n instances (observations) for Y and $\boldsymbol{X}$, $\{(y^{(1)}, \boldsymbol{x}^{(1)}), (y^{(2)}, \boldsymbol{x}^{(2)}), \ldots, (y^{(n)}, \boldsymbol{x}^{(n)})\}$ suffices to maximize the likelihood $\ell(\boldsymbol{\theta})$ in terms of parameters $\boldsymbol{\theta}$. The likelihood of n observations is defined by

$$\ell(\boldsymbol{\theta}) := \prod_{i=1}^{n} p_{\boldsymbol{\theta}}(\boldsymbol{x}^{(i)})^{y^{(i)}} (1 - p_{\boldsymbol{\theta}}(\boldsymbol{x}^{(i)}))^{1-y^{(i)}}. \tag{1}$$

For subgraph-subsequence pair (g, s), we can consider two explanatory variables X_1 and X_2 for subgraph g and subsequence s, respectively, each taking 1 if an observation has the corresponding substructure; otherwise zero. We use two logistic regression models for Y where $Y = 1$ for drug-target pairs (and $Y = 0$ for non-interacting pairs), i.e. the probability that $Y = 1$:

$$p_{\theta:0\text{-}2}(X_1, X_2) = \frac{\exp(\eta)}{1+\exp(\eta)} \quad \text{and} \quad p_{\theta:0\text{-}3}(X_1, X_2) = \frac{\exp(\eta + \theta_3 X_1 X_2)}{1+\exp(\eta + \theta_3 X_1 X_2)},$$

where $\eta = \theta_0 + \theta_1 X_1 + \theta_2 X_2$, and the second model has interaction term $\theta_3 X_1 X_2$ while the first model has no interaction terms. Parameters of these two models can be fitted by maximum likelihood estimation independently. Then, the significance of pair (g, s) is measured by testing whether $\theta_3 = 0$ is kept or not statistically. Note that this can be conducted by using the likelihood ratio test of two maximum likelihoods $\widehat{L}_{\theta:0\text{-}2}$ for $p_{\theta:0\text{-}2}(X_1, X_2)$ and $\widehat{L}_{\theta:0\text{-}3}$ for $p_{\theta:0\text{-}3}(X_1, X_2)$. The test statistic $-2\log(\widehat{L}_{\theta:0\text{-}2}/\widehat{L}_{\theta:0\text{-}3})$ follows the chi-squared distribution with one degree of freedom under the hypothesis that $\theta_3 = 0$. Thus, we can compute the p value of the observed statistic from the chi-squared distribution.

2.2 Computing likelihood ratio test numerically

We use the Newton-Raphson method to fit the logistic regression model to the data. Note that this method is a typical and standard manner for our problem setting. Explanatory variables X_1 and X_2, as well as response variable Y, are both binary, thus observations for (Y, X_1, X_2) can take only eight possible combinations because $Y \in \{0, 1\}, X_1 \in \{0, 1\}$ and $X_2 \in \{0, 1\}$, as shown in Table 1a. Thus, we need to count how many times each of eight combinations occurs in given observations.

In fact, we can compute the likelihood ratio test using logistic regression from the counts of eight possible combinations: P_{00}, P_{01}, P_{10}, P_{11}, N_{00}, N_{01}, N_{10}, and N_{11} in Table 1. Denoting $(X_1 = 0, X_2 = 0)$ by $\boldsymbol{x}_{00}$, the probability that $\boldsymbol{x}_{00}$ is

Table 1: Tables for counting eight values.

(a) model without the interaction term

explanatory			response	
	X_1	X_2	$\#\{Y=1\}$	$\#\{Y=0\}$
$\boldsymbol{x}_{00}$	0	0	P_{00}	N_{00}
$\boldsymbol{x}_{01}$	0	1	P_{01}	N_{01}
$\boldsymbol{x}_{10}$	1	0	P_{10}	N_{10}
$\boldsymbol{x}_{11}$	1	1	P_{11}	N_{11}

(b) model with the interaction term

explanatory				response	
	X_1	X_2	X_1X_2	$\#\{Y=1\}$	$\#\{Y=0\}$
$\boldsymbol{x}_{000}$	0	0	0	P_{00}	N_{00}
$\boldsymbol{x}_{010}$	0	1	0	P_{01}	N_{01}
$\boldsymbol{x}_{100}$	1	0	0	P_{10}	N_{10}
$\boldsymbol{x}_{111}$	1	1	1	P_{11}	N_{11}

observed P_{00} times (from observations with $Y=1$) and N_{00} times (from observations with $Y=0$) can be written:

$$\overbrace{p(\boldsymbol{x}_{00})\times\cdots\times p(\boldsymbol{x}_{00})}^{P_{00}}\times\overbrace{(1-p(\boldsymbol{x}_{00}))\times\cdots\times(1-p(\boldsymbol{x}_{00}))}^{N_{00}}=p(\boldsymbol{x}_{00})^{P_{00}}(1-p(\boldsymbol{x}_{00}))^{N_{00}},$$

and the entire likelihood $\ell(\boldsymbol{\theta})$ of Eq. (1) is implicitly given for binary variables Y, X_1, and X_2 by

$$p(\boldsymbol{x}_{00})^{P_{00}}(1-p(\boldsymbol{x}_{00}))^{N_{00}}p(\boldsymbol{x}_{01})^{P_{01}}(1-p(\boldsymbol{x}_{01}))^{N_{01}}p(\boldsymbol{x}_{00})^{P_{10}}(1-p(\boldsymbol{x}_{00}))^{N_{10}}p(\boldsymbol{x}_{11})^{P_{11}}(1-p(\boldsymbol{x}_{00}))^{N_{11}}.$$

Thus, letting an index set be $\Lambda:=\{00,01,10,11\}$, the log likelihood can be written:

$$\begin{aligned}L(\boldsymbol{\theta}) &:= \log\ell(\boldsymbol{\theta})=\log\left\{\prod_{\lambda\in\Lambda}p(\boldsymbol{x}_\lambda)^{P_\lambda}(1-p(\boldsymbol{x}_\lambda))^{N_\lambda}\right\}=\sum_{\lambda\in\Lambda}\{P_\lambda\log p(\boldsymbol{x}_\lambda)+N_\lambda\log(1-p(\boldsymbol{x}_\lambda))\}\\ &=\sum_{\lambda\in\Lambda}\left\{P_\lambda\log\frac{p(\boldsymbol{x}_\lambda)}{1-p(\boldsymbol{x}_\lambda)}+(P_\lambda+N_\lambda)\log(1-p(\boldsymbol{x}_\lambda))\right\},\end{aligned}$$

which means that we can compute the p value of likelihood ratio test using logistic regression by using only P_{00}, P_{01}, P_{10}, P_{11}, N_{00}, N_{01}, N_{01}, N_{11}. To maximize $L(\boldsymbol{\theta})$ by changing $\boldsymbol{\theta}$, the Newton-Raphson method repeats the following update:

$$\boldsymbol{\theta}^{[k+1]}\leftarrow\boldsymbol{\theta}^{[k]}+(\nabla^2L(\boldsymbol{\theta}))^{-1}\nabla L(\boldsymbol{\theta})\qquad\text{until}\quad\left|\frac{L(\boldsymbol{\theta}^{[k+1]})-L(\boldsymbol{\theta}^{[k]})}{L(\boldsymbol{\theta}^{[k]})}\right|<\epsilon,$$

where score $\nabla L(\boldsymbol{\theta})=\nabla\log\ell(\boldsymbol{\theta})$ and the Hessian matrix (asymptotic Fisher information matrix) are given as

$$\nabla L(\boldsymbol{\theta})=\begin{bmatrix}\partial L(\boldsymbol{\theta})/\partial\theta_0\\ \partial L(\boldsymbol{\theta})/\partial\theta_1\\ \partial L(\boldsymbol{\theta})/\partial\theta_2\end{bmatrix}=\begin{bmatrix}\sum_{\lambda\in\Lambda}(P_\lambda-(P_\lambda+N_\lambda)p(\boldsymbol{x}_\lambda))\\ \sum_{\lambda\in\Lambda}(x_1)_\lambda(P_\lambda-(P_\lambda+N_\lambda)p(\boldsymbol{x}_\lambda))\\ \sum_{\lambda\in\Lambda}(x_2)_\lambda(P_\lambda-(P_\lambda+N_\lambda)p(\boldsymbol{x}_\lambda))\end{bmatrix}$$

$$\nabla^2L(\boldsymbol{\theta})=\begin{bmatrix}\frac{\partial^2L(\boldsymbol{\theta})}{\partial\theta_0\theta_0}&\frac{\partial^2L(\boldsymbol{\theta})}{\partial\theta_0\theta_1}&\frac{\partial^2L(\boldsymbol{\theta})}{\partial\theta_0\theta_2}\\ \frac{\partial^2L(\boldsymbol{\theta})}{\partial\theta_1\theta_0}&\frac{\partial^2L(\boldsymbol{\theta})}{\partial\theta_1\theta_1}&\frac{\partial^2L(\boldsymbol{\theta})}{\partial\theta_1\theta_2}\\ \frac{\partial^2L(\boldsymbol{\theta})}{\partial\theta_2\theta_0}&\frac{\partial^2L(\boldsymbol{\theta})}{\partial\theta_2\theta_1}&\frac{\partial^2L(\boldsymbol{\theta})}{\partial\theta_2\theta_2}\end{bmatrix},\quad\frac{\partial^2L(\boldsymbol{\theta})}{\partial\theta_i\theta_j}=\sum_{\lambda\in\Lambda}(P_\lambda+N_\lambda)(x_i)_\lambda(x_j)_\lambda p(\boldsymbol{x}_\lambda)(1-p(\boldsymbol{x}_\lambda)).$$

Hence, the Newton-Raphson update can be written in a matrix form:

$$\boldsymbol{\theta}^{[k+1]}\leftarrow\boldsymbol{\theta}^{[k]}+(\boldsymbol{X}'\boldsymbol{W}\boldsymbol{X})^{-1}\boldsymbol{X}'(\boldsymbol{y}-\boldsymbol{p}),\tag{2}$$

where when we use a logistic model $p_{\theta:0\text{-}2}(\cdot)$ as $p(\cdot)$,

$$\boldsymbol{X}:=\begin{bmatrix}1&0&0\\1&0&1\\1&1&0\\1&1&1\end{bmatrix},\quad\boldsymbol{W}:=\begin{bmatrix}d_{00}&0&0&0\\0&d_{01}&0&0\\0&0&d_{10}&0\\0&0&0&d_{11}\end{bmatrix},\quad\boldsymbol{y}:=\begin{bmatrix}P_{00}\\P_{01}\\P_{10}\\P_{11}\end{bmatrix},\quad\boldsymbol{p}:=\begin{bmatrix}(P_{00}+N_{00})\,p_{\theta:0\text{-}2}(\boldsymbol{x}_{00})\\(P_{01}+N_{01})\,p_{\theta:0\text{-}2}(\boldsymbol{x}_{01})\\(P_{10}+N_{10})\,p_{\theta:0\text{-}2}(\boldsymbol{x}_{10})\\(P_{11}+N_{11})\,p_{\theta:0\text{-}2}(\boldsymbol{x}_{11})\end{bmatrix},$$

$$d_{00} = (P_{00} + N_{00})\, p_{\theta:0\text{-}2}(\boldsymbol{x}_{00})(1 - p_{\theta:0\text{-}2}(\boldsymbol{x}_{00})), \qquad d_{01} = (P_{01} + N_{01})\, p_{\theta:0\text{-}2}(\boldsymbol{x}_{01})(1 - p_{\theta:0\text{-}2}(\boldsymbol{x}_{01})),$$
$$d_{10} = (P_{10} + N_{10})\, p_{\theta:0\text{-}2}(\boldsymbol{x}_{10})(1 - p_{\theta:0\text{-}2}(\boldsymbol{x}_{10})), \qquad d_{11} = (P_{11} + N_{11})\, p_{\theta:0\text{-}2}(\boldsymbol{x}_{11})(1 - p_{\theta:0\text{-}2}(\boldsymbol{x}_{11})),$$

and when we use a logistic model $p_{\theta:0\text{-}3}(\cdot)$ as $p(\cdot)$,

$$\boldsymbol{X} := \begin{bmatrix} 1 & 0 & 0 & 0 \\ 1 & 0 & 1 & 0 \\ 1 & 1 & 0 & 0 \\ 1 & 1 & 1 & 1 \end{bmatrix}, \quad \boldsymbol{W} := \begin{bmatrix} d_{000} & 0 & 0 & 0 \\ 0 & d_{010} & 0 & 0 \\ 0 & 0 & d_{100} & 0 \\ 0 & 0 & 0 & d_{111} \end{bmatrix}, \quad \boldsymbol{y} := \begin{bmatrix} P_{00} \\ P_{01} \\ P_{10} \\ P_{11} \end{bmatrix}, \quad \boldsymbol{p} := \begin{bmatrix} (P_{00} + N_{00})\, p_{\theta:0\text{-}3}(\boldsymbol{x}_{000}) \\ (P_{01} + N_{01})\, p_{\theta:0\text{-}3}(\boldsymbol{x}_{010}) \\ (P_{10} + N_{10})\, p_{\theta:0\text{-}3}(\boldsymbol{x}_{100}) \\ (P_{11} + N_{11})\, p_{\theta:0\text{-}3}(\boldsymbol{x}_{111}) \end{bmatrix},$$

$$d_{000} = (P_{00} + N_{00})\, p_{\theta:0\text{-}3}(\boldsymbol{x}_{000})(1 - p_{\theta:0\text{-}3}(\boldsymbol{x}_{000})), \qquad d_{010} = (P_{01} + N_{01})\, p_{\theta:0\text{-}3}(\boldsymbol{x}_{010})(1 - p_{\theta:0\text{-}3}(\boldsymbol{x}_{010})),$$
$$d_{100} = (P_{10} + N_{10})\, p_{\theta:0\text{-}3}(\boldsymbol{x}_{100})(1 - p_{\theta:0\text{-}3}(\boldsymbol{x}_{100})), \qquad d_{111} = (P_{11} + N_{11})\, p_{\theta:0\text{-}3}(\boldsymbol{x}_{111})(1 - p_{\theta:0\text{-}3}(\boldsymbol{x}_{111})).$$

The deviance for maximum likelihood $\hat{\boldsymbol{\theta}}$ is defined by

$$D := -2(L(\hat{\boldsymbol{\theta}}) - L^*)$$

where L^* is the log likelihood by the so-called full model (or saturated model) where probabilities can be given in the following:

$$p(\boldsymbol{x}_{00}) = \frac{P_{00}}{P_{00} + N_{00}}, \quad p(\boldsymbol{x}_{01}) = \frac{P_{01}}{P_{01} + N_{01}}, \quad p(\boldsymbol{x}_{10}) = \frac{P_{10}}{P_{10} + N_{10}}, \quad p(\boldsymbol{x}_{11}) = \frac{P_{11}}{P_{11} + N_{11}},$$

resulting in that the log likelihood L^* can be obtained as

$$L^* = \sum_{\lambda \in \Lambda} \left\{ P_\lambda \log \frac{P_\lambda}{P_\lambda + N_\lambda} + N_\lambda \log \frac{N_\lambda}{P_\lambda + N_\lambda} \right\}.$$

The deviance thus can be written by

$$D(\hat{\boldsymbol{\theta}}) = 2(L^* - L(\hat{\boldsymbol{\theta}})) = 2 \cdot \sum_{\lambda \in \Lambda} \left\{ P_\lambda \log \frac{P_\lambda}{(P_\lambda + N_\lambda) p_{\hat{\boldsymbol{\theta}}}(\boldsymbol{x}_\lambda)} + N_\lambda \log \frac{N_\lambda}{(P_\lambda + N_\lambda)(1 - p_{\hat{\boldsymbol{\theta}}}(\boldsymbol{x}_\lambda))} \right\}.$$

2.3 Computing quantities for likelihood ratio test efficiently

In our algorithm, each time we found a frequent subgraph-subsequence pair (g, s) in $\mathcal{Q}$, we evaluate the statistical significance of (g, s) by the Newton-Raphson update in Eq. (2). So far we have shown that this computation is realized by computing only eight values: P_{00}, P_{01}, P_{10}, P_{11}, N_{00}, N_{01}, N_{10}, and N_{11}, which are shown in Table 1 for the likelihood-ratio test in our setting. In our experiment, we used all possible combinations of drugs and targets, i.e. $\mathcal{G}_\mathcal{Q}$ and $\mathcal{S}_\mathcal{Q}$, for non-interacting pairs. Under this setting, we present an efficient manner of computing the counts of the eight combinations for this case.

Given subgraph-subsequence pair (g, s), set $\mathcal{G}_\mathcal{Q}$ can be divided into graphs $\mathcal{G}_g$ which contain g and $\mathcal{G}_{\bar{g}}$ which do not contain g. Similarly, sequence set $\mathcal{S}_\mathcal{Q}$ can be divided into sequences $\mathcal{S}_s$ which have s and $\mathcal{S}_{\bar{s}}$ which do not have s (Fig. 4). This means that each of all graph-sequence pairs $\mathcal{G}_\mathcal{Q} \times \mathcal{S}_\mathcal{Q}$ can be fallen into one of the following eight types:

1. $\mathcal{P}_{11} := \{(G, S) \in \mathcal{Q} \mid g \in G, s \in S\}$,
2. $\mathcal{N}_{11} := \{(G, S) \in \mathcal{G}_\mathcal{Q} \times \mathcal{S}_\mathcal{S} \mid g \in G, s \in S, (G, S) \notin \mathcal{Q}\}$,
3. $\mathcal{P}_{01} := \{(G, S) \in \mathcal{Q} \mid g \notin G, s \in S\}$,
4. $\mathcal{N}_{01} := \{(G, S) \in \mathcal{G}_\mathcal{Q} \times \mathcal{S}_\mathcal{S} \mid g \notin G, s \in S, (G, S) \notin \mathcal{Q}\}$,
5. $\mathcal{P}_{10} := \{(G, S) \in \mathcal{Q} \mid g \in G, s \notin S\}$,
6. $\mathcal{N}_{10} := \{(G, S) \in \mathcal{G}_\mathcal{Q} \times \mathcal{S}_\mathcal{S} \mid g \in G, s \notin S, (G, S) \notin \mathcal{Q}\}$,
7. $\mathcal{P}_{00} := \{(G, S) \in \mathcal{Q} \mid g \notin G, s \notin S\}$,
8.. $\mathcal{N}_{00} := \{(G, S) \in \mathcal{G}_\mathcal{Q} \times \mathcal{S}_\mathcal{S} \mid g \notin G, s \notin S, (G, S) \notin \mathcal{Q}\}$.

and from these sets, we can have $P_{00} = |\mathcal{P}_{00}|$, $P_{01} = |\mathcal{P}_{01}|$, $P_{10} = |\mathcal{P}_{10}|$, $P_{11} = |\mathcal{P}_{11}|$, $N_{00} = |\mathcal{N}_{00}|$, $N_{01} = |\mathcal{N}_{01}|$, $N_{10} = |\mathcal{N}_{10}|$, and $N_{11} = |\mathcal{N}_{11}|$.

The algorithm shown in Proposition 6 first builds enumeration tree $\mathcal{T}_g$ for frequent subgraphs in terms of the marginal support. For each subgraph $g \in \mathcal{T}_g$, we can thus compute support$(g \mid \mathcal{G}_\mathcal{Q}) = |\mathcal{G}_g|$ and support$((g, *) \mid \mathcal{Q}) = P_{11} + P_{10}$. Then the algorithm traverses $\mathcal{T}_s$ and for each $s \in \mathcal{T}_s$, we choose $g \in \mathcal{T}_g$ in a depth-first manner and test the significance of (g, s). Thus, we have $\mathcal{S}_s$ and $\{(G, S) \in \mathcal{Q} \mid s \subseteq S\}$ which is $\mathcal{P}_{11} + \mathcal{P}_{01}$. Furthermore since we have g, if $g \subseteq G$, we can see that pair (G, S) in $\{(G, S) \in \mathcal{Q} \mid s \subseteq S\}$ is in $\mathcal{P}_{11}$; otherwise it is in $\mathcal{P}_{01}$. Overall at this moment, we can have the following values regarding pair (g, s):

1. $|\mathcal{G}_\mathcal{Q}|$, $|\mathcal{S}_\mathcal{Q}|$, and $|\mathcal{Q}|$.

2. $|\mathcal{G}_g|$ and $|\mathcal{S}_s|$.

3. support$((g, *) \mid \mathcal{Q}) = P_{11} + P_{10}$

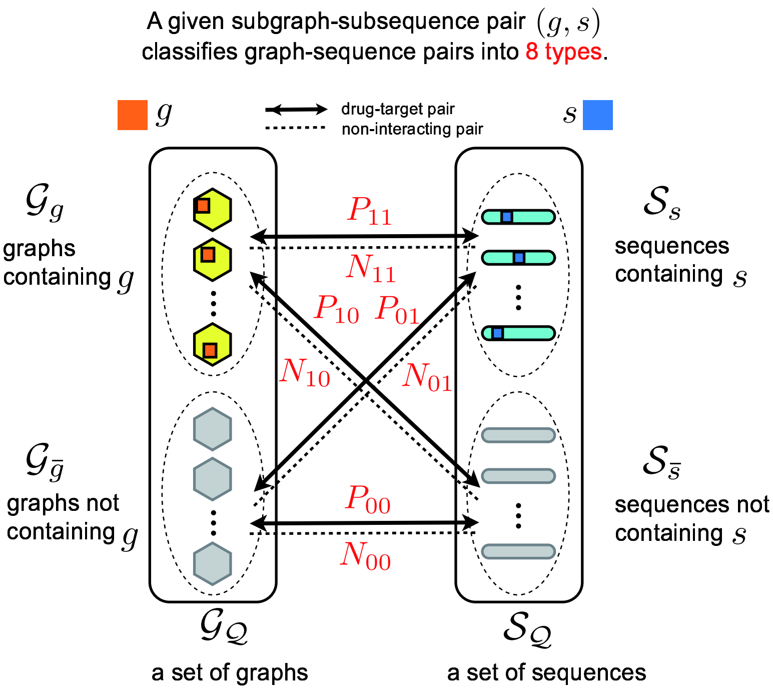

Figure 4: Division of graph sets $\mathcal{G}_{\mathcal{Q}}$ and sequence sets $\mathcal{S}_{\mathcal{Q}}$ by a subgraph-subsequence pair (g, s).

4. P_{11} and P_{01}.

From these values, we can compute the remaining six values: P_{10}, P_{00}, N_{00}, N_{01}, N_{10}, N_{11}.

1. $P_{10} = (P_{11} + P_{10}) - P_{11}$
2. $N_{11} = |\mathcal{G}_g| \times |\mathcal{G}_s| - P_{11}$.
3. $N_{01} = (|\mathcal{G}_{\mathcal{Q}}| - |\mathcal{G}_g|) \times |\mathcal{G}_s| - P_{01}$.
4. $N_{10} = |\mathcal{G}_g| \times (|\mathcal{S}_{\mathcal{Q}}| - |\mathcal{G}_s|) - P_{10}$.
5. $P_{00} = |\mathcal{Q}| - P_{01} - P_{10} - P_{11}$.
6. $N_{00} = (|\mathcal{G}_{\mathcal{Q}}| - |\mathcal{G}_g|) \times (|\mathcal{S}_{\mathcal{Q}}| - |\mathcal{G}_s|) - P_{00}$.

3 Finding substructure pairs in given graph-sequence pairs exactly and efficiently

Once we obtained significant substructure pairs, for an arbitrary given compound-protein pair, we can generate a binary vector where an element is 1 if the corresponding substructure pair is in the given pair; otherwise zero. We call this vector a GRASP fingerprint, by which any compound-protein pair can be characterized by significant substructure pairs. A GRASP fingerprint is very useful, since we can check the similarity between two compound-protein pairs by comparing their GRASP fingerprints.

To obtain the GRASP fingerprint for a given compound-protein pair, we need to test whether or not each substructure pair is in the given compound-protein pair. This means that we have to solve two problems: subgraph isomorphism and subsequence (substring, exactly) matching. In the work, we used 10,000 most significant substructure pairs, meaning that we have to solve these two problems 10,000 times for a given compound-protein pair. If you take straight-forward approaches to solve the two problems, it is infeasible to compute GRASP fingerprints of a large-scale compound-protein pairs in a practical computation time. In fact, in the work, we used 975,143,103 pairs (generated from 140,937 compounds and 6,919 proteins) for which the number of combinations reaches approximately 9.5×10^{12}. We then developed an efficient approach for this issue, considering the following four properties:

Property 1: A large-scale compound-protein pairs can be a bipartite graph. Checking all edges independently is redundant, since a lot of edges share the same graphs or the same strings.

Property 2: Similarly, significant substructure pairs can be also given as a bipartite graph on subgraphs and subsequences.

Property 3: Subgraphs (subsequences) in significant substructure pairs often include smaller subgraphs (subsequences) in other substructure pairs. Thus, a lot of subgraph isomorphism problems to be solved are strongly related with each other, even if subgraphs are different. This is true of subsequences.

Property 4: We can obtain canonical representations of subgraphs and subsequences in our algorithm, meaning that a subgraph can be represented as an 'edge sequence', which is called the minimum DFS code [2]. This makes our problem easier.

Properties 1 and 2 lead us to take the following strategy: We first decompose given graph-string pairs into a set of graphs and a set of sequences. Similarly, a set of subgraphs and a set of subsequences are generated from significant substructure pairs. We then test whether subgraphs (subsequences) are in graphs (sequences) or not, and integrate this information by using original two bipartite graphs on graph-sequence pairs and significant substructure pairs.

A problem in this strategy is the method to test whether subgraphs are in graphs and whether subsequences are in sequences. Since we can use a common technique to these two cases and a graph can be represented by a minimum DFS code, which is a sequence, we hereafter do not distinguish between graphs and sequences and explain only either of them appropriately.

Properties 3 and 4 provide us an efficient method to solve this problem. First Property 4 implies an efficient manner to test whether a subgraph is in a graph, since a graph can be an "edge sequence" or a minimum DFS code in some order. That is, intuitively if the minimum DFS code of a subgraph is in the minimum DFS code of a graph, we can say that the subgraph is in the graph. Usually this manner can be fast, since an input subgraph is generally not in a graph. Next the minimum DFS code of Property 4 realizes the idea of Property 3. For example, assume that we have three sequences (a,b,c,d), (a,b,c,e), (a,b,d). The most straightforward way to test whether each of these sequences is in given sequences or not is testing these sequences one by one. However, this can be made faster by using the following idea: After being checked by (a,b,c,d), we can skip testing the first three letters, i.e. (a,b,c), of (a,b,c,e), because (a,b,c,d) and (a,b,c,e) share (a,b,c), i.e. a common prefix sequence. Similarly we can skip the first two letters of (a,b,d), since they are already examined. This example is on sequences, but graphs can be edge sequences or minimum DFS codes, and so this can be exactly the case with graphs. Note that this idea already implements Property 3. In fact, this manner can be systematically implemented by using a compressed tree structure called 'Patricia tree' (or 'Radix tree'), in which common prefix sequences of minimum DFS codes are stored at nodes. Fig. 5 shows an example of representing given sequences by a Patricia tree. You can see that by traversing each node of this tree only once, we can check all sequences without duplication. Note that similar to enumeration trees, a longer sequence is at a node in a deeper level, meaning that the downward closure property works on a Patricia tree. That is, sequences at more descendant nodes appear in a smaller number of instances.

Overall Fig. 6 shows toy examples on the entire procedure we can develop. Fig. 6a shows our problem in which the input is two sets, i.e. graph-sequence pairs and subgraph-subsequence pairs, in the left-hand side, and the output is the matrix in the right-hand side, where a circle shows that the corresponding graph-sequence pair has the corresponding subgraph-subsequence pair. We first decompose the input into a set of graphs (and subgraphs) and a set of sequences (and subsequences). We then build two Patricia trees, one for subgraphs in Fig. 6b and the other for subsequences in Fig. 6c. Next instances are traversed over these trees as in Fig. 6d and 6e, resulting in that we can see graphs (sequences) that contain subgraphs (subsequences). Lastly we integrate subgraphs with subsequences, according to the bipartite graph of subgraphs and subsequences (Fig. 6f), and further map them into the bipartite graph of graphs and sequences (Fig. 6g). We finally obtain the exact information on graph-sequence pairs which contain subgraph-subsequence pairs.

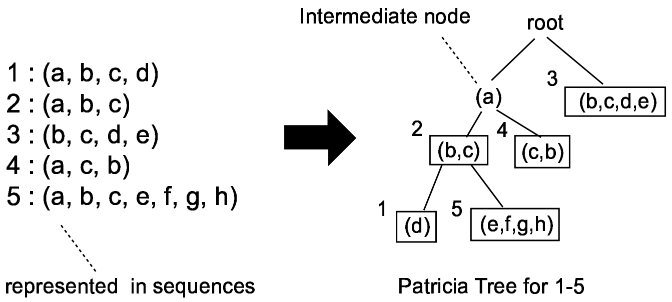

Figure 5: An example of a Patricia tree. For example, given five sequences in the left-hand side, they are stored at nodes of the tree in the right-hand side. Note that each sequence corresponds to a path from the root to the corresponding node.

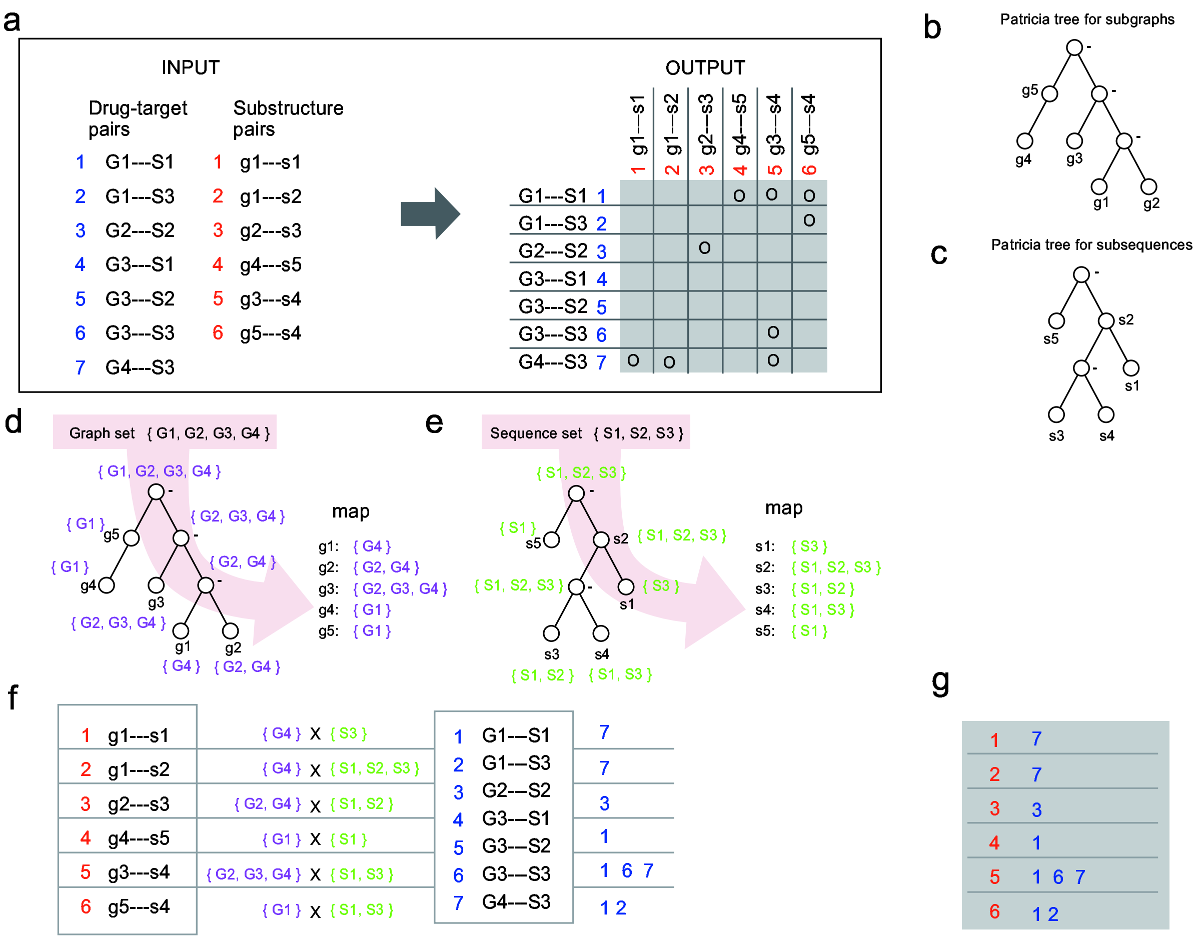

Figure 6: The entire procedure for matching substructure pairs to given graph-sequence pairs exactly and efficiently.

4 Finding the most similar graph-sequence pair in terms of GRASP fingerprints exactly and efficiently

Let u be a drug-target pair and $\mathcal{D} = \{u_1, u_2, \ldots, u_{|\mathcal{D}|}\}$ be a set of drug-target pairs. For a compound-protein pair v, we are interested in finding the pair in $\mathcal{D}$ that has the most similar GRASP fingerprint to v. That is, our problem here is that for arbitrary given v, we develop an efficient manner for comparing v with $u \in \mathcal{D}$ in terms of GRASP fingerprints.

Given n significant substructure pairs, which can be defined as a subset of $\{1, 2, \ldots, n\}$, the GRASP fingerprint of graph-sequence pair v shows that substructure pairs contained in v. We denote the GRASP fingerprint of graph-sequence pair v by $f(q) \subset \{1, 2, \ldots, n\}$. We can measure the similarity of u and v by Tanimoto coefficient (Tc) of two GRASP fingerprints $f(u)$ and $f(v)$:

$$\mathrm{Tc}(u, v) := \frac{|f(u) \cap f(v)|}{|f(u) \cup f(v)|}.$$

In reality, this amount can be efficiently computed by (# of 1s in $\mathrm{bit}(u) \wedge \mathrm{bit}(v)$) / (# of 1s in $\mathrm{bit}(u) \vee \mathrm{bit}(v)$), where $\mathrm{bit}(\cdot)$ is a vector of n bits and $\wedge$ and $\vee$ denote bit-AND and bit-OR operations, respectively. Using these notations, finding $u \in \mathcal{D}$ with the most similar GRASP fingerprint to that of given v can be the following nearest neighbor search problem

$$\hat{u} := \arg\max_{u \in \mathcal{D}} \mathrm{Tc}(u, v). \tag{3}$$

Under the setting of $n = 10,000, |\mathcal{D}| = 11,219$ in the work, we had to solve 975,143,103 nearest neighbor search problems. Thus, again, we need an efficient procedure for avoiding unnecessary evaluations.

Note here that the simple upper bound of Tc,

$$\mathrm{Tc}(u, v) \leqslant \frac{|f(u) \cap f(v)|}{\max(f(u), f(v))} \leqslant \frac{\min(f(u), f(v))}{\max(f(u), f(v))},$$

has the monotonically decreasing property given by

$$\text{if } |f(x)| \leqslant |f(u)| \leqslant |f(v)| \text{ or } |f(x)| \geqslant |f(u)| \geqslant |f(v)|, \text{ then } \frac{\min(f(x), f(u))}{\max(f(x), f(v))} \geqslant \frac{\min(f(x), f(v))}{\max(f(x), f(v))},$$

we can derive the following efficient method to solve Eq. (3) [4].

Proposition 7 (Efficient nearest neighbor search in terms of Tc)**.**

1. *Sort* $\mathcal{D}$ *so that* $|f(u_1)| \leqslant |f(u_2)| \leqslant \cdots \leqslant |f(u_{|\mathcal{D}|})|$.
2. *Compute* $|f(v)|$ *and find* i*, satisfying* $|f(u_i)| \leqslant |f(v)| \leqslant |f(u_{i+1})|$*, by using a binary search.*
3. *Compute* $\mathrm{Tc}(v, u_j)$ *for* $j = i+1, i+2, \ldots, |\mathcal{D}|$ *and for* $j = i, i-1, i-2, \ldots, 1$ *in order so that* $b_j := \frac{\min(f(x), f(u_j))}{\max(f(x), f(u_j))}$ *decreases monotonically. Let* m_j *be the maximum Tc in the computed values by the* j*-th step.*
4. *When* $m_j > b_j$ *holds, terminate the search. The value* b_j *is the decreasing upper bound of* $\mathrm{Tc}(v, u_j)$*, thus* $m_j > b_j$ *means no larger Tc than current* m_j *exist for further* j*. The pair* $\hat{u}$ *that gives* $m_j = \mathrm{Tc}(v, \hat{u})$ *is the nearest neighbor to given* v*.*

References

[1] J. Pei, J. Han, B. Mortazavi-Asl, H. Pinto, Q. Chen, U. Dayal, and M. Hsu, Prefixspan: Mining sequential patterns by prefix-projected growth. *IEEE International Conference on Data Engineering (ICDE'01)*, 215–224, 2001.

[2] X. Yan and J. Han, gSpan: Graph-based substructure pattern mining. *IEEE International Conference on Data Mining (ICDM'02)*, 721–724, Washington, DC, USA, 2002.

[3] H. Cordell, Epistasis, What it means, what it doesn't mean, and statistical methods to detect it in humans. *Hum. Mol. Genet.* 11, 2463–8, 2002.

[4] S. J. Swamidass and P. Baldi, Bounds and algorithms for fast exact searches of chemical fingerprints in linear and sublinear time.*J. Chem. Inf. Model.*, 47 (2):302–317, 2007.
