# Supplementary material for: Mining Significant Substructure Pairs for Interpreting Polypharmacology in Drug-Target Network
Source: PLoS One. 2011 Feb 23;6(2):e16999. doi: 10.1371/journal.pone.0016999 (PMC3044142; doi:10.1371/journal.pone.0016999)
Supplement: Table S1 — For each of R1 to R8, (a) the number of drug-target pairs, (b) the number of drug-target pairs which gave the highest Tc of larger than 0.95 for pairs in POSI and (c) the number of drug-target pairs which gave the highest Tc of larger than 0.95 for pairs in MASS. (PDF) [file pone.0016999.s006.pdf]

Table S1: For each of R1 to R8, (a) the number of drug-target pairs, (b) the number of drug-target pairs which gave the highest Tc of larger than 0.95 for pairs in POSI and (c) the number of drug-target pairs which gave the highest Tc of larger than 0.95 for pairs in MASS.

	R1	R2	R3	R4	R5	R6	R7	R8	other	Total
#drug-target pairs	283 (2.52%)	287 (2.56%)	1,848 (16.47%)	180 (1.60%)	555 (4.95%)	465 (4.14%)	222 (1.98%)	741 (6.60%)	6,638 (59.17%)	11,219 (100%)
#drug-target pairs with highest Tc > 0.95 for POSI	17 (7.30%)	6 (2.58%)	3 (1.29%)	2 (0.86%)	7 (3.00%)	11 (4.72%)	0 (0%)	40 (17.17%)	147 (63.09%)	233 (100%)
#drug-target pairs with highest Tc > 0.95 for MASS	9,865 (0.85%)	29,696 (2.56%)	928,957 (79.97%)	186 (0.016%)	129 (0.011%)	134 (0.012%)	147,531 (12.70%)	10,874 (0.93%)	34,241 (2.95%)	1,161,613 (100%)
