# Supplementary material for: Mining Significant Substructure Pairs for Interpreting Polypharmacology in Drug-Target Network
Source: PLoS One. 2011 Feb 23;6(2):e16999. doi: 10.1371/journal.pone.0016999 (PMC3044142; doi:10.1371/journal.pone.0016999)
Supplement: Table S2 — The number of drug-target pairs with the number of drugs and targets in each of R1 to R8. (PDF) [file pone.0016999.s007.pdf]

Table S2: The number of drug-target pairs with the number of drugs and targets in each of R1 to R8.

Cluster	R1	R2	R3	R4	R5	R6	R7	R8
#pairs (#drugs, #targets)	283 (154, 149)	287 (136, 204)	1848 (840, 540)	180 (63, 146)	555 (138, 406)	465 (135, 333)	222 (88,84)	741 (219, 413)
#pairs (#drugs, #targets) of promiscuous drugs only	205 (76, 111)	216 (65, 178)	1451 (443, 462)	140 (23, 125)	508 (91, 393)	419 (89, 324)	176 (42, 66)	621 (99, 379)
