# Supplementary material for: Mining Significant Substructure Pairs for Interpreting Polypharmacology in Drug-Target Network
Source: PLoS One. 2011 Feb 23;6(2):e16999. doi: 10.1371/journal.pone.0016999 (PMC3044142; doi:10.1371/journal.pone.0016999)
Supplement: Table S3 — The most related 30 GO terms with each of R1 to R8. The most right column shows the value of the number of genes in both the corresponding cluster and the corresponding GO term divided by the number of genes in the corresponding GO term. (PDF) [file pone.0016999.s008.pdf]

Table S3: The most related 30 GO terms with each of R1 to R8. The most right column shows the value of the number of genes in both the corresponding cluster and the corresponding GO term divided by the number of genes in the corresponding GO term.

R1

	P value	GO	Description	Rate
molecular function	1.17e-05	0004879	ligand-dependent nuclear receptor activity	11/30
	3.7e-05	0003707	steroid hormone receptor activity	10/28
	3.7e-05	0043565	sequence-specific DNA binding	11/35
	5.17e-05	0005496	steroid binding	9/23
	0.000433	0003700	transcription factor activity	11/46
	0.000433	0004872	receptor activity	27/257
	0.0016	0004957	prostaglandin E receptor activity	4/5
	0.0016	0004953	icosanoid receptor activity	5/9
	0.0016	0004954	prostanoid receptor activity	5/9
	0.00486	0003924	GTPase activity	7/25
	0.00486	0015926	glucosidase activity	3/3
	0.00486	0004558	alpha-glucosidase activity	3/3
	0.00535	0003677	DNA binding	13/86
	0.00585	0004955	prostaglandin receptor activity	4/7
	0.00831	0008289	lipid binding	13/91
	P value	GO	Description	Rate
biological process	2.62e-06	0007018	microtubule-based movement	8/11
	3.78e-06	0030705	cytoskeleton-dependent intracellular transport	8/12
	3.7e-05	0007017	microtubule-based process	8/16
	7.0e-05	0051258	protein polymerization	7/13
	0.00278	0008610	lipid biosynthetic process	13/79
	0.00535	0044255	cellular lipid metabolic process	19/175
	0.0058	0007010	cytoskeleton organization and biogenesis	9/44
	0.0101	0032774	RNA biosynthetic process	13/94
	0.0101	0006351	transcription, DNA-dependent	13/94
	P value	GO	Description	Rate
cellular component	0.000157	0005874	microtubule	8/20
	0.0016	0044430	cytoskeleton#cytoskeletal part	9/36
	0.0016	0015630	microtubule cytoskeleton	8/28
	0.00452	0005856	cytoskeleton	11/61
	0.0085	0042598	vesicular fraction	9/47
	0.0085	0005792	microsome	9/47

R2

	P value	GO	Description	Rate
molecular function	0.00258	0020037	heme binding	11/39
	0.00258	0046906	tetrapyrrole binding	11/39
	0.0134	0005506	iron ion binding	16/103
	0.0307	0016709	oxidoreductase activity, acting on paired donors, with incorporation or reduction of molecular oxygen, NADH or NADPH as one donor, and incorporation of one atom of oxygen	5/10
	0.0318	0004745	retinol dehydrogenase activity	4/6
	0.0318	0005497	androgen binding	3/3
	0.0413	0008236	serine-type peptidase activity	9/39
	0.0413	0017171	serine hydrolase activity	9/40
	-0.0413	0016740	transferase activity	5/322
	-0.0503	0032555	purine ribonucleotide binding	4/285
	-0.0503	0032553	ribonucleotide binding	4/285
	0.0523	0016705	oxidoreductase activity, acting on paired donors, with incorporation or reduction of molecular oxygen	10/53
	0.0523	0004252	serine-type endopeptidase activity	8/36
	0.0557	0008233	peptidase activity	13/90
	0.0584	0004954	prostanoid receptor activity	4/9
	0.0584	0004953	icosanoid receptor activity	4/9
	0.0596	0004175	endopeptidase activity	11/66
	0.0634	0004879	ligand-dependent nuclear receptor activity	7/30
	P value	GO	Description	Rate
biological process	0.000159	0006775	fat-soluble vitamin metabolic process	8/13
	0.00258	0006776	vitamin A metabolic process	6/10
	0.0413	0065008	regulation of biological quality	23/195
	0.0441	0006766	vitamin metabolic process	9/41
	0.0469	0050878	regulation of body fluid levels	9/42
	0.0469	0050817	coagulation	8/34
	0.0469	0007596	blood coagulation	8/34
	0.0523	0007599	hemostasis	8/36
	0.0584	0006720	isoprenoid metabolic process	4/9
	0.0584	0001763	morphogenesis of a branching structure	4/9
cellular component	P value	GO	Description	Rate
	0.0318	0044421	extracellular region#extracellular region part	15/101
	0.0523	0005615	extracellular space	13/89

R3

	P value	GO	Description	Rate
molecular function	8.41e-26	0004872	receptor activity	139/257
	4.17e-25	0004871	signal transducer activity	158/313
	4.17e-25	0060089	molecular transducer activity	158/313
	1.52e-23	0001584	rhodopsin-like receptor activity	69/95
	1.76e-23	0004888	transmembrane receptor activity	109/188
	6.16e-21	0004930	G-protein coupled receptor activity	72/107
	4.38e-19	0008227	amine receptor activity	34/35
	8.27e-16	0022836	gated channel activity	62/97
	1.22e-14	0015075	ion transmembrane transporter activity	86/160
	5.37e-14	0046873	metal ion transmembrane transporter activity	52/79
	5.37e-14	0022803	passive transmembrane transporter activity	64/107
	5.37e-14	0015267	channel activity	64/107
	7.09e-14	0005216	ion channel activity	63/105
	1.43e-13	0022838	substrate specific channel activity	63/106
	2.86e-13	0005261	cation channel activity	48/72
	4.11e-13	0022843	voltage-gated cation channel activity	35/45
	P value	GO	Description	Rate
biological process	2.87e-19	0007166	cell surface receptor linked signal transduction	128/254
	5.45e-19	0007186	G-protein coupled receptor protein signaling pathway	86/145
	1.03e-15	0007154	cell communication	208/523
	8.93e-15	0019932	second-messenger-mediated signaling	56/86
	9.75e-14	0007165	signal transduction	182/452
	7.25e-13	0006811	ion transport	85/165
	P value	GO	Description	Rate
cellular component	1.44e-28	0005886	plasma membrane	212/453
	2.59e-28	0044459	plasma membrane part	179/357
	2.36e-27	0005887	integral to plasma membrane	149/278
	2.36e-27	0031226	intrinsic to plasma membrane	149/278
	3.24e-25	0016021	integral to membrane	223/509
	4.17e-25	0031224	intrinsic to membrane	223/510
	3.02e-18	0044425	membrane#membrane part	243/624
	8.27e-16	0016020	membrane	275/760

R4

	P value	GO	Description	Rate
molecular function	3.24e-57	0048037	cofactor binding	45/111
	3.12e-52	0030170	pyridoxal phosphate binding	42/44
	8.79e-32	0019842	vitamin binding	43/89
	9.89e-27	0016769	transferase activity, transferring nitrogenous groups	25/29
	5.57e-22	0008483	transaminase activity	20/22
	3.3e-09	0016829	lyase activity	21/69
	1.27e-07	0005231	excitatory extracellular ligand-gated ion channel activity	13/29
	2.88e-06	0004972	N-methyl-D-aspartate selective glutamate receptor activity	6/6
	2.88e-06	0004889	nicotinic acetylcholine-activated cation-selective channel activity	6/6
	3.05e-06	0016831	carboxy-lyase activity	9/16
	3.6e-06	0005230	extracellular ligand-gated ion channel activity	13/38
	1.03e-05	0016740	transferase activity	38/322
	2.15e-05	0043176	amine binding	10/25
	2.34e-05	0016830	carbon-carbon lyase activity	9/20
	3.81e-05	0022834	ligand-gated channel activity	13/47
	3.81e-05	0015276	ligand-gated ion channel activity	13/47
	9.28e-05	0016846	carbon-sulfur lyase activity	6/9
	P value	GO	Description	Rate
biological process	7.9e-07	0006519	amino acid and derivative metabolic process	29/195
	8.87e-06	0006807	nitrogen compound metabolic process	30/224
	2.34e-05	0009308	amine metabolic process	28/209
	6.27e-05	0009069	serine family amino acid metabolic process	8/17
	7.84e-05	0006520	amino acid metabolic process	23/163
	0.000237	0019752	carboxylic acid metabolic process	31/266
	0.000255	0006082	organic acid metabolic process	31/267
	P value	GO	Description	Rate
cellular component	2.88e-06	0044456	synapse#synapse part	15/50
	7.11e-06	0045211	postsynaptic membrane	14/47
	7.84e-05	0043235	receptor complex	9/23
	0.000142	0030054	cell junction	15/70
	0.00036	0008328	integral to plasma membrane#ionotropic glutamate receptor complex	4/4
	0.00036	0017146	N-methyl-D-aspartate selective glutamate receptor complex	4/4

R5

	P value	GO	Description	Rate
molecular function	1.9e-11	0000166	nucleotide binding	62/322
	5.6e-11	0030554	adenyl nucleotide binding	54/267
	8.88e-10	0017076	purine nucleotide binding	57/305
	2.06e-09	0032559	adenyl ribonucleotide binding	49/247
	2.65e-08	0032555	purine ribonucleotide binding	52/285
	2.65e-08	0032553	ribonucleotide binding	52/285
	3.71e-06	0005524	ATP binding	43/239
	6.99e-05	0016208	AMP binding	6/6
	0.000779	0016740	transferase activity	48/322
	0.00152	0009055	electron carrier activity	19/88
	0.00256	0016301	kinase activity	26/143
	0.00322	0016772	transferase activity, transferring phosphorus-containing groups	28/161
	0.006	0008146	sulfotransferase activity	5/7
	0.00903	0016773	phosphotransferase activity, alcohol group as acceptor	23/130
	0.0141	0030551	cyclic nucleotide binding	4/5
	P value	GO	Description	Rate
biological process	8.71e-05	0005975	carbohydrate metabolic process	27/132
	0.0008	0044262	cellular carbohydrate metabolic process	22/106
	0.00322	0055086	nucleobase, nucleoside and nucleotide metabolic process	16/71
	0.00322	0006164	purine nucleotide biosynthetic process	9/22
	0.00758	0006163	purine nucleotide metabolic process	9/25
	0.00758	0006091	generation of precursor metabolites and energy	29/178
	0.0103	0044237	cellular metabolic process	106/1011
	0.0154	0016310	phosphorylation	26/159
	P value	GO	Description	Rate
cellular component	0.000893	0005622	intracellular	110/1010
	0.00447	0044424	intracellular#intracellular part	106/990
	-0.00581	0005887	integral to plasma membrane	6/278
	-0.00581	0031226	intrinsic to plasma membrane	6/278
	0.00653	0044446	intracellular organelle#intracellular organelle part	48/350
	0.00758	0044422	organelle#organelle part	48/353
	-0.0229	0044459	plasma membrane part	13/357

R6

	P value	GO	Description	Rate
molecular function	1.48e-14	0016614	oxidoreductase activity, acting on CH-OH group of donors	24/75
	2.42e-11	0016491	oxidoreductase activity	51/302
	2.14e-10	0050662	coenzyme binding	23/61
	6.91e-10	0016616	oxidoreductase activity, acting on the CH-OH group of donors, NAD or NADP as acceptor	24/71
	6.8e-08	0048037	cofactor binding	24/111
	P value	GO	Description	Rate
biological process	1.74e-11	0044262	cellular carbohydrate metabolic process	27/106
	2.42e-11	0019318	hexose metabolic process	24/60
	2.42e-11	0005996	monosaccharide metabolic process	24/61
	4.9e-10	0006066	alcohol metabolic process	26/110
	4.9e-10	0006096	glycolysis	15/25
	5.56e-09	0005975	carbohydrate metabolic process	28/132
	1.07e-08	0046365	monosaccharide catabolic process	15/30
	1.07e-08	0019320	hexose catabolic process	15/30
	1.07e-08	0006007	glucose catabolic process	15/30
	1.81e-08	0046164	alcohol catabolic process	15/31
	4.38e-07	0006006	glucose metabolic process	17/49
	6.19e-07	0009057	macromolecule catabolic process	18/75
	6.66e-07	0044275	cellular carbohydrate catabolic process	15/39
	9.57e-07	0016052	carbohydrate catabolic process	15/40
	5.51e-06	0044265	cellular macromolecule catabolic process	16/51
	1.68e-05	0006091	generation of precursor metabolites and energy	29/178
	P value	GO	Description	Rate
cellular component	1.31e-07	0043233	organelle lumen	25/121
	1.31e-07	0031974	membrane-enclosed lumen	25/121
	5.71e-06	0044446	intracellular organelle#intracellular organelle part	47/350
	7.84e-06	0044422	organelle#organelle part	47/353
	1.05e-05	0005737	cytoplasm	88/885
	-1.33e-05	0031224	intrinsic to membrane	10/510
	-1.36e-05	0016021	integral to membrane	10/509
	1.46e-05	0044429	mitochondrial part	27/159
	1.9e-05	0044424	intracellular#intracellular part	94/990

R7

	P value	GO	Description	Rate
molecular function	2.38e-25	0008227	amine receptor activity	24/35
	9.94e-19	0004888	transmembrane receptor activity	31/188
	1.16e-16	0001584	rhodopsin-like receptor activity	27/95
	3.17e-15	0004930	G-protein coupled receptor activity	27/107
	1.49e-12	0004872	receptor activity	32/257
	9.85e-10	0042165	neurotransmitter binding	14/38
	1.04e-09	0004871	signal transducer activity	33/313
	1.04e-09	0060089	molecular transducer activity	33/313
	6.2e-09	0004935	adrenoceptor activity	8/10
	6.77e-08	0030594	neurotransmitter receptor activity	12/35
	1.12e-07	0004936	alpha-adrenergic receptor activity	6/6
	4.08e-07	0042166	acetylcholine binding	7/10
	2.68e-06	0004981	muscarinic acetylcholine receptor activity	5/5
	6.99e-06	0015464	acetylcholine receptor activity	6/9
	P value	GO	Description	Rate
biological process	1.12e-20	0007186	G-protein coupled receptor protein signaling pathway	28/145
	1.46e-10	0007166	cell surface receptor linked signal transduction	30/254
	9.85e-10	0003008	system process	30/266
	1.54e-06	0019932	second-messenger-mediated signaling	16/86
	7.59e-06	0007187	G-protein signaling, coupled to cyclic nucleotide second messenger	12/52
	9.2e-06	0019935	cyclic-nucleotide-mediated signaling	12/53
	1.25e-05	0007188	G-protein signaling, coupled to cAMP nucleotide second messenger	10/36
	P value	GO	Description	Rate
cellular component	1.12e-20	0005887	integral to plasma membrane	40/278
	1.12e-20	0031226	intrinsic to plasma membrane	40/278
	2.4e-18	0044459	plasma membrane part	44/357
	5.62e-15	0005886	plasma membrane	47/453
	6.96e-13	0016021	integral to membrane	48/509
	7.12e-13	0031224	intrinsic to membrane	48/510
	2.77e-12	0044425	membrane#membrane part	53/624
	3.12e-09	0016020	membrane	55/760
	8.84e-07	0005624	membrane fraction	21/180

R8

	P value	GO	Description	Rate
molecular function	2.71e-10	0030246	carbohydrate binding	22/50
	3.06e-06	0005529	sugar binding	13/27
	3.06e-06	0004553	hydrolase activity, hydrolyzing O-glycosyl compounds	12/23
	2.73e-05	0016798	hydrolase activity, acting on glycosyl bonds	12/28
	0.000175	0017171	serine hydrolase activity	13/40
	0.000193	0016758	transferase activity, transferring hexosyl groups	9/19
	0.00073	0008236	serine-type peptidase activity	12/39
	0.000816	0008378	galactosyltransferase activity	5/6
	0.000816	0035250	UDP-galactosyltransferase activity	5/6
	0.000889	0030247	polysaccharide binding	9/23
	0.000931	0016787	hydrolase activity	42/326
	0.00144	0008233	peptidase activity	17/90
	0.00144	0003945	N-acetyllactosamine synthase activity	4/4
	0.00144	0005537	mannose binding	4/4
	0.00157	0001871	pattern binding	9/25
	P value	GO	Description	Rate
biological process	1.53e-09	0005975	carbohydrate metabolic process	30/132
	6.31e-06	0006955	immune response	19/81
	6.31e-06	0002376	immune system process	23/110
	2.88e-05	0009611	response to wounding	23/117
	0.000103	0009605	response to external stimulus	28/166
	0.000298	0007599	hemostasis	12/36
	0.000816	0042060	wound healing	12/40
	0.000816	0050817	coagulation	11/34
	0.000816	0007596	blood coagulation	11/34
	0.00119	0050878	regulation of body fluid levels	12/42
	P value	GO	Description	Rate
cellular component	2.73e-05	0044421	extracellular region#extracellular region part	21/101
	5.11e-05	0005615	extracellular space	19/89
	0.000103	0005764	lysosome	13/38
	0.000103	0000323	lytic vacuole	13/38
	0.000135	0005773	vacuole	13/39
