# Supplementary material for: Mining Significant Substructure Pairs for Interpreting Polypharmacology in Drug-Target Network
Source: PLoS One. 2011 Feb 23;6(2):e16999. doi: 10.1371/journal.pone.0016999 (PMC3044142; doi:10.1371/journal.pone.0016999)
Supplement: Table S4 — For each of R1 to R8, the average Tc of paired GRASP fingerprints over all drug-target pairs in the corresponding cluster, and that over 105 clusters, each having interactions randomly selected out of the original 11,219 drug-target pairs and keeping the cluster size the same as that of the corresponding cluster. (PDF) [file pone.0016999.s009.pdf]

Table S4: For each of R1 to R8, the average Tc of paired GRASP fingerprints over all drug-target pairs in the corresponding cluster, and that over 10^5 clusters, each having interactions randomly selected out of the original 11,219 drug-target pairs and keeping the cluster size the same as that of the corresponding cluster.

Cluster	R1	R2	R3	R4	R5	R6	R7	R8
Average Tc of GRASP fingerprints	0.3174	0.2590	0.3566	0.2426	0.3944	0.3474	0.3822	0.4765
Average Tc of GRASP fingerprints over 10^5 random clusters (RC)	0.03739	0.03741	0.03741	0.03742	0.03741	0.03742	0.03741	0.03741
Standard deviation of RC	0.00317	0.00316	0.00114	0.00402	0.00222	0.00245	0.00360	0.00191
Maximum of RC	0.05384	0.05503	0.04278	0.06011	0.04791	0.05117	0.05659	0.04716
