# Supplementary material for: Mining Significant Substructure Pairs for Interpreting Polypharmacology in Drug-Target Network
Source: PLoS One. 2011 Feb 23;6(2):e16999. doi: 10.1371/journal.pone.0016999 (PMC3044142; doi:10.1371/journal.pone.0016999)
Supplement: Table S7 — In each of R1 to R8, given drugs and targets of drug-target pairs (of promiscuous drugs), the ratio of drug-target pairs which were in the corresponding cluster to all drug-target pairs between them, and the average over those of 105 clusters, each having drug-target pairs (of promiscuous drugs) randomly selected out of the original 11,219 drug-target interactions and keeping the cluster size the same as that of the corresponding cluster. (PDF) [file pone.0016999.s012.pdf]

Table S7: In each of R1 to R8, given drugs and targets of drug-target pairs (of promiscuous drugs), the ratio of drug-target pairs which were in the corresponding cluster to all drug-target pairs between them, and the average over those of 10^5 clusters, each having drug-target pairs (of promiscuous drugs) randomly selected out of the original 11,219 drug-target interactions and keeping the cluster size the same as that of the corresponding cluster.

Cluster	R1	R2	R3	R4	R5	R6	R7	R8
Ratio	1.00 (205/205)	0.9153 (216/236)	0.9932 (1451/1461)	0.9655 (140/145)	0.9621 (508/528)	0.9790 (419/428)	1.00 (176/176)	0.9920 (621/626)
Average ratio by 10^5 random clusters (RC)	0.5679	0.5664	0.4986	0.6185	0.5127	0.5245	0.5938	0.4976
Standard deviation of RC	0.0536	0.0533	0.0146	0.0696	0.0349	0.0392	0.0617	0.0286
Max of RC	0.68	0.678	0.532	0.788	0.594	0.614	0.735	0.560
