# Supplementary material for: Mining Significant Substructure Pairs for Interpreting Polypharmacology in Drug-Target Network
Source: PLoS One. 2011 Feb 23;6(2):e16999. doi: 10.1371/journal.pone.0016999 (PMC3044142; doi:10.1371/journal.pone.0016999)
Supplement: Table S8 — The number of drug-target pairs sharing the same drugs and the average sequence identity between target (amino acid sequences) of these pairs. (PDF) [file pone.0016999.s013.pdf]

Table S8: The number of drug-target pairs sharing the same drugs and the average sequence identity between targets (amino acid sequences) of these pairs

Cluster	R1	R2	R3	R4	R5	R6	R7	R8	Average
#pairs of drug-target pairs sharing the same drugs	327	3,828	2,761	3,107	6,554	5,254	495	13,405	
Average sequence identity between targets (drug-target pairs) sharing the same drug	0.3128	0.0212	0.2371	0.0372	0.0127	0.0148	0.2784	0.0927	0.0311
